# A conserved Y-shaped RNA structure in the 3’UTR of chikungunya virus genome as a host-specialized element that modulates viral replication and evolution

**DOI:** 10.1371/journal.ppat.1011352

**Published:** 2023-05-01

**Authors:** Eugenia Soledad Bardossy, Sebastiano Volpe, Diego Ezequiel Alvarez, Claudia Verónica Filomatori

**Affiliations:** 1 Instituto de Investigaciones Biotecnológicas, Universidad Nacional de San Martín—CONICET, Buenos Aires, Argentina; 2 Escuela de Bio y Nanotecnología, Universidad de San Martín, Buenos Aires, Argentina; University of California Los Angeles, UNITED STATES

## Abstract

RNA viral genomes compact information into functional RNA structures. Here, using chikungunya virus as a model, we investigated the structural requirements of conserved RNA elements in the 3’ untranslated region (3’UTR) for viral replication in mosquito and mammalian cells. Using structural predictions and co-variation analysis, we identified a highly stable and conserved Y-shaped structure (SLY) at the end of the 3’UTR that is duplicated in the Asian lineage. Functional studies with mutant viruses showed that the SLY has host-specific functions during viral replication and evolution. The SLY positively modulates viral replication in mosquito cells but has the opposite effect in mammalian cells. Additional structural/functional analyses showed that maintaining the Y-shaped fold and specific nucleotides in the loop are critical for full SLY functionality and optimal viral replication in mosquito cells. Experimental adaptation of viruses with duplicated SLYs to mammalian cells resulted in the generation of heterogeneous viral populations comprising variants with diverse 3’UTRs, contrasting with the homogeneous populations from viruses without SLY copies. Altogether, our findings constitute the first evidence of an RNA secondary structure in the 3’UTR of chikungunya virus genome that plays host-dependent functions.

## Author summary

Chikungunya virus (CHIKV) is a re-emergent and rapidly spreading virus transmitted from mosquitoes to humans. To jump between hosts, the virus has to evolve strategies to efficiently replicate using different cellular machineries and mount mechanisms to evade host-specific antiviral responses. Here, we identified an RNA motif at the 3’ end of the viral genome that folds into a Y-shaped structure and is present in CHIKV epidemic strains. Through a systematic mutational study, we discovered that this RNA motif enhances viral replication in mosquito cells but has the opposite effect in mammalian cells. Moreover, we observed that an intact overall structure and specific loop sequences are crucial for optimal viral replication in the mosquito host. Importantly, in vitro evolution experiments showed that duplication of this sequence, as found in Asian lineage strains, generates diverse CHIKV variants within the viral population grown in mammalian cells. Our findings represent the first report of a cis-acting RNA motif in the CHIKV genome’s 3’ untranslated region (CHIKV 3’UTR) whose functions during viral replication and evolution differs between hosts.

## Introduction

Viruses have extremely compact genomes and have developed strategies to optimize their use [1,2]. One strategy is the presence of functional RNA structures within both coding and non-coding regions that are important for modulating viral replication and adaptation to hosts [3–7]. Many cellular and viral biological processes are highly dependent on specific RNA structures and/or sequences [3,8]. Generally, viral RNA elements crucial for viral replication are highly conserved, often constraining the ability of viruses to evolve [9] and evade the immune response [2,10]. Moreover, some RNA structures contain overlapping RNA signals that modulate different viral processes [11,12] and in the case of vector-borne viruses might be subject to opposite selective pressures in the alternate hosts. Therefore, RNA structures usually drive viral evolution and they are target of selection [2]. Our knowledge of how virus-host coevolution influences viral RNA sequences and structures remains poor. Here, we used an RNA virus that alternates between invertebrate and vertebrate hosts as a model for studying the role of conserved RNA elements during viral replication and adaptation to mammalian and insect cells.

Chikungunya virus (CHIKV) is an RNA virus transmitted to humans via the bite of infected mosquitoes that has recently re-emerged and spread globally [13]. In humans, it causes a febrile syndrome accompanied by joint pain that can persist for months or years, accounting for most of the global CHIKV-associated disease burden [14]. There is no antiviral drug therapy to treat CHIKV or a licensed vaccine to prevent its infection. Therefore, understanding the underlying molecular mechanisms that govern CHIKV biology is fundamental for developing strategies to mitigate expanding CHIKV epidemics.

CHIKV is a small and enveloped virus belonging to the *Alphavirus* genus in the *Togaviridae* family. It has a non-segmented, positive sense, single-stranded RNA genome of 11–12 kb in length with a type 0 cap (N7mGppp-) at the 5’ end and a poly(A) tail at the 3’ end. The viral genome contains two open reading frames encoding nonstructural and structural polyproteins. The 5’ and 3’ untranslated regions (UTRs) are located at the ends of the viral genome. The 5’UTR is short (76 nt) and highly conserved, while the 3’UTR varies greatly in length (500–900 nt) and sequence organization between natural virus isolates [6].

The 5’ and 3’ ends of viral genomes often modulate RNA translation and synthesis of RNA plus and minus strands. A combination of linear sequences and structural RNA elements usually govern these processes. For example, the 5’ terminal dinucleotide AU is highly conserved in alphaviruses and is believed to be important for initiating plus-strand RNA synthesis [15,16]. In addition, an RNA stem-loop structure at the 5’ terminus of the alphavirus 5’UTR has been proposed to promote minus-strand RNA synthesis [15,16] and is involved in avoiding recognition by the cellular innate immune response in mammalian cells [10]. Also, several conserved RNA structural elements within the non-structural protein 1 (nsP1) coding region have roles in viral genome replication, through human/mosquito host-dependent and independent mechanisms [17].

Characterizing conserved structural elements in the alphaviruses’ 3’UTR has remained challenging due to its appreciable variability among different species and strains of the same species. However, most alphavirus 3’UTRs share a variable number of short repeated sequence elements (RSEs). The RSEs in the CHIKV 3’UTR are commonly named direct repeats (DRs) and occur in lineage-specific patterns that likely resulted from historical duplication events [6,18,19]. We have recently investigated the role of DRs during CHIKV replication and evolution, finding that they are subject to opposite selective pressures in different hosts [20]. DRs enhance replication in the mosquito host in vitro and in vivo, while they are redundant in mammalian cells. Indeed, viral variants with shorter 3’UTRs are widely generated by RNA recombination and positively selected in mammalian cells. When the virus passes to mosquitoes, variants with short 3’UTRs are negatively selected, and those with an intact 3’UTR prevail. Based on these findings, we have proposed a model to explain the 3’UTR function during host switch in which genomic recombination acts together with natural selection to shape the spectrum of 3’UTR variants in the viral population, enabling the virus to cross species barriers efficiently [20].

While we now better understand the role of DRs during CHIKV transmission cycle, knowledge about functional RNA structures in the CHIKV 3’UTR is still missing. Regarding DR folding into RNA structural elements, we and others have reported that the last part of the CHIKV 3’UTR forms a highly stable and conserved Y-shaped stem-loop (SLY hereafter), recently confirmed by biochemical probing of the CHIKV’s complete genome in solution [18,20,21]. Additionally, a recent phylogenetic analysis suggested an association between CHIKV DRs and distinct RNA secondary structures conserved among virus lineages [22]. While several pieces of evidence support SLY formation in solution, there is no data on its precise role during CHIKV replication and evolution.

To investigate SLY’s function we have rigorously analyzed the RNA elements in the 3’UTR of different CHIKV isolates belonging to the Indian Ocean (IOL), East Central and South Africa (ECSA), and Asian epidemic lineages. We have also characterized SLY’s sequence and structural traits in detail and studied its role during CHIKV replication and adaptation to host cells. Combined sequence conservation and covariance analyses strongly confirmed a highly stable and conserved SLY at the end of the CHIKV 3’UTR. Using reverse genetics, we showed that this structural RNA element enhances viral replication in mosquito cells but is dispensable in mammalian cells. Additional functional studies revealed that maintaining the Y-shape folding is crucial for infectivity in mosquito cells since disruption of base pairings significantly compromised viral replication. Finally, host-cell adaptation experiments in mammalian cells uncovered that RNA recombination occurs at high rates at the CHIKV 3’UTR regardless of the SLY. Nevertheless, the presence or absence of SLY structure/s modulated the composition of CHIKV viral populations during in vitro evolution, probably due to selective pressures acting on the viral genome’s 3’UTR. Altogether, our results represent the first evidence that a conserved RNA element in the CHIKV 3’UTR has an important role during viral replication and evolution in a host-dependent manner.

## Results

### Identification of a conserved RNA secondary structure in the CHIKV 3’UTR

**Fig 1:**
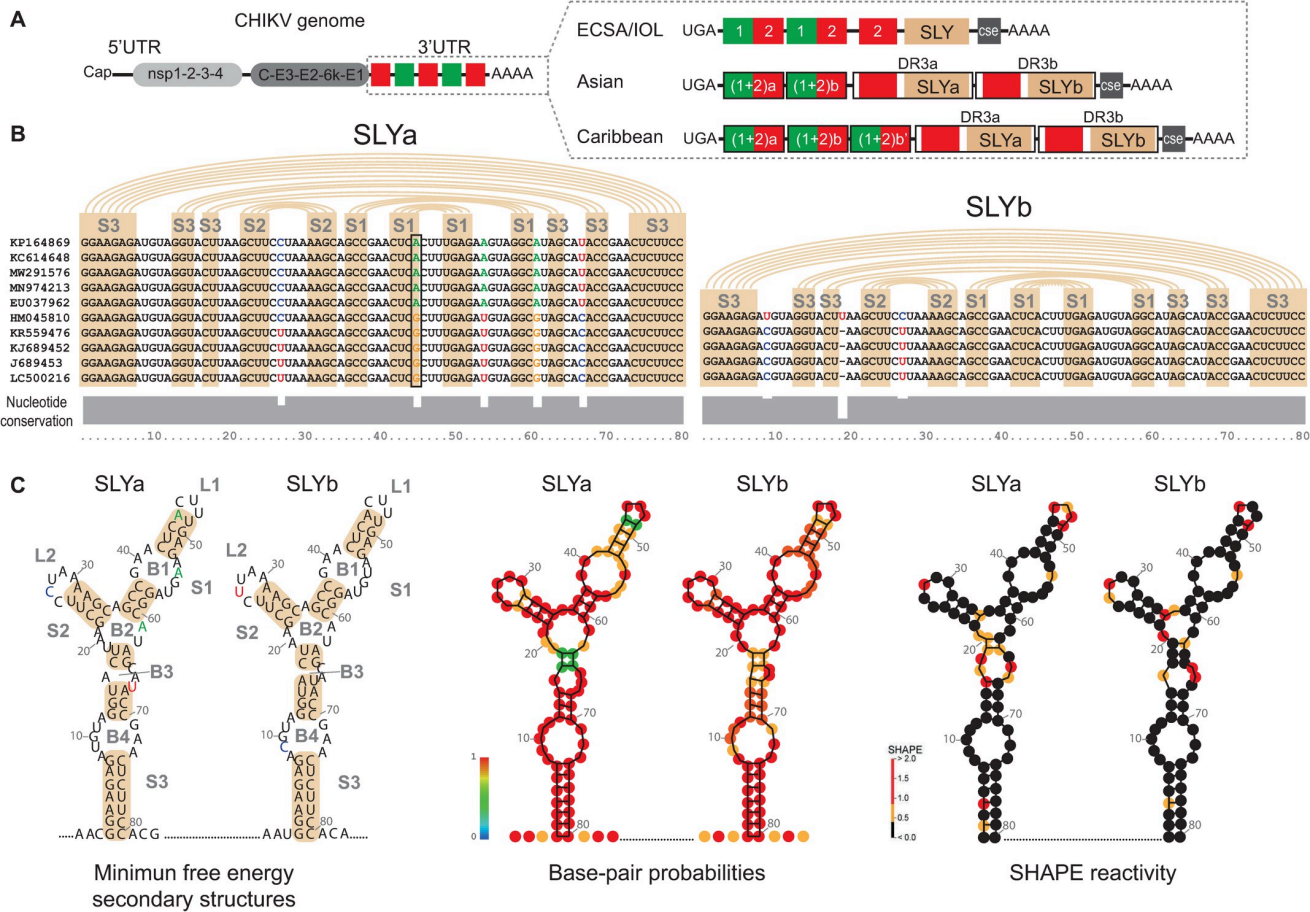
RNA secondary structures in the CHIKV 3’UTR. (A) Schematic representation of the genomic organization of CHIKV (left) and its 3’UTR (right) for the ECSA, IOL, and Asian epidemic lineages and the Caribbean strain. Colored boxes represent different DRs. The DR3 region corresponding to SLY is colored in pink. (B) Sequence alignment (middle) and nucleotide conservation plot (bottom, in gray) of the 80 nt-long DR3 region that folds into SLYa and SLYb, for ten representative ECSA, IOL, and Asian lineages isolates. Pink arcs and boxes indicate the nucleotide pairings that form the stems of each Y. Conserved nucleotides are in black, and variable nucleotides are in red, blue, green and orange. The black box indicates the nucleotide position 45/49 that covariates among lineages. (C) Consensus RNA secondary structures predicted for SLYa and SLYb. Left, minimum-free energy foldings with the corresponding consensus sequences. Middle, base-pair probabilities. For unpaired regions, the color represents the probability of being unpaired. Right, the SHAPE-constrained thermodynamic model for SLYa and SLYb. Nucleotide positions are numbered every 10 nucleotides. SLY, stem-loop Y; S1, stem 1; S2, stem 2; S3, stem 3; L1, loop 1; L2, loop 2; B1, bulge 1; B2, bulge 2; B3, bulge 3; B4, bulge 4.

The CHIKV 3’UTR contains DRs that occur in lineage-specific patterns [6,18]. The four major CHIKV lineages are the epidemic viruses of the IOL, ECSA, and Asian lineages and the enzootic virus of the Western African lineage. The genomes of the IOL, ECSA and West African lineages contain shorter 3’UTRs with two DR element types (DR1, two copies; and DR2, three copies). In contrast, the Asian lineage contains a longer 3’UTR that has accumulated insertions and point mutations around DR1 and DR2 [referred to as DR (1+2)], and the duplication of an entirely new region (DR3) [18]. Interestingly, some American CHIKV isolates derived from the Asian lineage, such as those in the Caribbean, contain an additional 177 nucleotide (nt) duplication [DR (1+2)b’], displaying the longest 3’UTR described among CHIKV isolates (Fig 1A) [23].

To identify conserved RNA secondary structures in the CHIKV 3’UTR, we first aligned the 3’UTR sequences of 112 natural isolates from the IOL, ECSA and Asian epidemic lineages, and used RNA folding analysis to predict their 3’UTR secondary structures. We identified at the end of the 3’UTR an 80 nt-long region within DR3a and DR3b that folds into a stable Y-shaped RNA structure (SLY henceforth). (Fig 1B and 1C). The SLY structure was present in a single copy in the 3’UTRs of ECSA and IOL lineages but duplicated in the Asian lineage. Each SLY comprises three stems: stem 1 (S1) exposes a right loop (L1) and is interrupted by one bulge (B1); stem 2 (S2) exposes a left loop (L2); and stem 3 (S3) leaves a central bulge (B2) and is interrupted by two other bulges (B3 and B4) (Fig 1C, left). SLYa and SLYb are highly stable and have similar predicted minimum free energies (−36.48 and −35.46 kcal/mol, respectively). In addition, according to modeling, base-pair probabilities for nucleotides forming both SLYs are high (Fig 1C, middle).

Next, we analyzed the SLY’s structure and nucleotide conservation, finding that almost all nucleotides involved in base-pairing are conserved (Fig 1B, bottom plot and Fig 1C, left). However, sequence variation appears at unpaired nucleotides forming loops or bulges (Fig 1B, bottom plot). For example, five nucleotides in the SLYa sequence varied among the analyzed isolates: one in a loop (nt 27 in L2), three in bulges (nt 54 in B1, nt 61 in B2, and nt 67 in B3), and one (nt 45) in S1. Half of the analyzed sequences had an adenosine at position 45, pairing uracil in position 49. In contrast, the other half had guanosine, maintaining the pairing with U49. The preservation of the overall SLY structure despite this nucleotide variation represents strong evidence for S1 formation. In SLYb, all nucleotides in the stems were completely conserved, and there were only three positions where nucleotides varied: one in a loop (nt 27 in L2) and two in bulges (nt 9 in B4 and nt 20 in B2).

The RNA secondary structure of the complete genome of a human CHIKV isolate from a 2013 outbreak on a Caribbean island (GenBank accession number MT228631) was recently mapped in solution using the selective 2′-hydroxyl acylation analyzed by primer extension (SHAPE)-mutational profiling technique [21]. The joint analysis of the 3’UTR sequence and its SHAPE reactivity showed that virus stocks comprise viral variants with differences in their 3’UTRs. These variants differ in length and RNA structures downstream the translation stop codon but had a common sequence and secondary structure at the end of the 3’UTR. To evaluate whether their secondary structure in solution supports the SLYa and SLYb folding, we thermodynamically modeled SLYa and SLYb using the SHAPE reactivity values published by Madden et al. as constraints [21] (Fig 1C, right). For both SLYs, nucleotides in S1, S2 and S3 consistently showed low reactivity values (less than 0.3, in black), suggesting that they are in non-flexible zones of the RNA and likely involved in base pairings. Notably, single-stranded B1, B4 and L1 terminal nucleotides of SLB were also poorly reactive, indicating they are likely involved in RNA tertiary interactions. In contrast, nucleotides in L1, L2, B2 and B3 had higher SHAPE values, indicating that these regions are exposed in the tertiary RNA structure.

Altogether, our analyses support the folding of a stable SLY in the CHIKV 3’UTR that is duplicated in the Asian lineage.

### The presence of a duplicated SLY contributes to 3’UTR variability after viral adaptation to mammalian cells

In a previous study, we followed the in vitro evolution of CHIKV populations, finding that new viral variants with deletions in their 3’UTRs emerge during adaptation to cells [20]. Specifically, restricting the virus to a single cell type resulted in 3’UTR variants with clean deletions of DR copies that arose with higher frequency in BHK mammalian cells than in C6/36 mosquito cells. Interestingly, many emergent variants contained only one DR3 copy in their 3’UTRs, and therefore a single SLY element. These observations raise questions about why Asian strains have retained a duplicated SLY in their 3’UTRs and whether SLY has a host-dependent function during the CHIKV life cycle.

**Fig 2:**
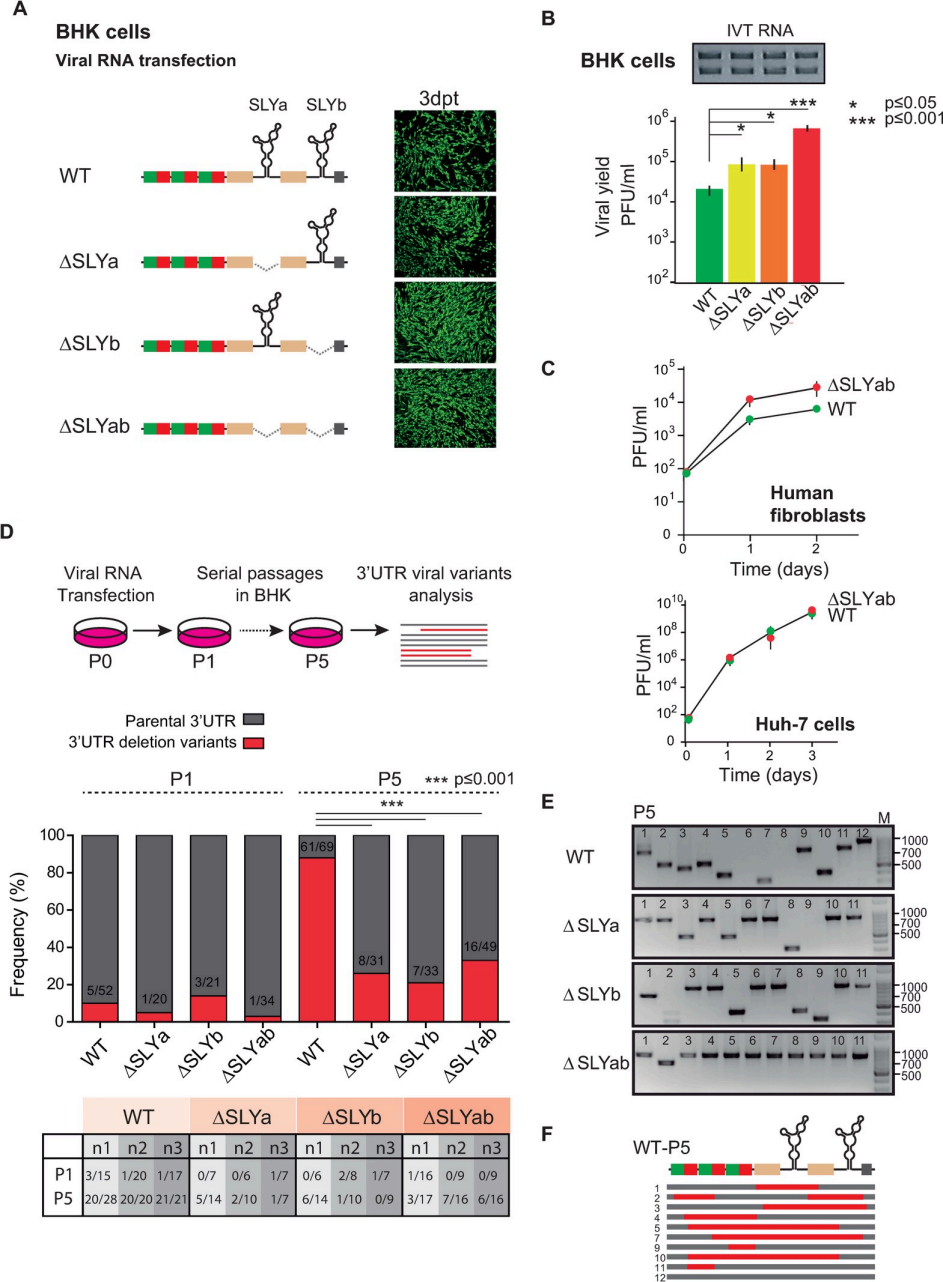
Replication and adaptation to mammalian cells of CHIKV-Caribbean and mutant viruses with SLY deletions. (A) Left, schematic representations of the 3’UTRs of the WT virus and mutant viruses with deletions of one or two SLY copies (ΔSLYa, ΔSLYb and ΔSLYab). Right, immunofluorescence stainings for WT and mutant viruses in mammalian BHK cells on day 3 post-transfection. Images correspond to one representative experiment out of three biological replicates. (B) Viral yields in cell culture supernatant on day 3 post-transfection for WT and mutant viruses. The symbols and bars depict the means ± standard deviations of the means from three independent experiments. Data were compared with a two-tailed, unpaired *t*-test. Top, in vitro transcribed (IVT) RNAs used for transfection in the experiments shown in Figs 2 and 4. (C) Comparative growth kinetics for WT and ΔSLYab viruses in human fibroblasts and Huh-7 cells. Cells were infected with MOI = 0.1 and data were compared with a two-tailed, unpaired *t*-test. The symbols and bars depict the means ± standard deviations of the means from two independent experiments. (D) Analysis of the 3’UTRs of the WT and mutant viruses restricted to replication in mammalian cells. Top, schematic representation of viral passages and analysis of the 3’UTRs. Middle, bar graphs showing the frequencies of viral variants in WT and mutant viral populations P1 and P5 in mammalian cells. The ratio of 3’UTR deletion variants to total clones is indicated inside the bars and corresponds to the cumulative data from three independent experiments. Data were compared pairwise with Fisher’s exact test on cumulative data. Bottom, table with the proportion of 3’UTR deletion variants from three independent experiments (n1, n2 and n3) in the WT, ΔSLYa, ΔSLYb and ΔSLYab populations. (E) Representative agarose gels for PCR amplification of the 3’UTRs of individual clones recovered from WT and mutant viral populations passaged five times in BHK cells. The sizes of DNA bands in the ladder (base pairs) are indicated on the right. (F) Schematic representation of the alignment of the 3’UTRs of viral variants comprising the WT P5 population. Red lines indicate deletions within viral variants. The corresponding sequence for each variant is shown in S1 Fig.

To investigate SLY’s role during CHIKV replication and evolution, we designed recombinant viruses in which we individually deleted one (ΔSLYa or ΔSLYb) or both (ΔSLYab) SLYs from the wild type (WT) CHIKV-Caribbean 3’UTR (Fig 2A, left). First, we evaluated the replication of WT and SLY deletion mutants in mammalian cells. To this end, we synthesized full-length viral RNAs by in vitro transcription (IVT) that were transfected in equal amounts (Fig 2B, top) into BHK cells. Then, we followed viral replication as a function of time through the detection of viral antigens and titration of infectious viral particles in cell culture supernatants. We observed that deleting either or both SLYs did not negatively affect viral replication in mammalian cells. WT and mutant (ΔSLYa, ΔSLYb and ΔSLYab) viruses could infect the entire cell monolayer after three days of transfection and caused extended cytopathic effect by day 6 (Fig 2A). The quantification of viral yields in culture supernatants showed that viruses with SLY deletions had an advantage when replicating in BHK cells: ΔSLYab, ΔSLYa and ΔSLYb reached viral titers 35-, four- and four-fold higher than those reached by the WT virus on day 3. To further characterize the ΔSLYab mutant phenotype in human cells, we infected fibroblasts and Huh-7 cells with the ΔSLYab and WT viruses. Growth curves showed that both viruses replicated similarly, despite a slight advantage of the ΔSLYab over the WT in human fibroblasts (Fig 2C). These results clearly indicate that the presence of SLY in the CHIKV 3’UTR is not necessary for efficient viral replication in mammalian cells, and surprisingly, the ΔSLYab deletion might have a positive effect on viral yields.

Second, we studied SLY’s role during CHIKV experimental evolution. We focused our analysis on the 3’UTR and investigated the composition of viral populations after the infection with the WT virus and mutant viruses carrying SLY deletions. Briefly, WT, ΔSLYa, ΔSLYb and ΔSLYab viral stocks recovered from transfection supernatants were serially passaged in mammalian BHK cells. Total RNA was extracted from culture supernatants for each viral population and used as a template for reverse transcription reactions with an oligo(dT) primer. The pool of viral cDNAs was used to amplify fragments corresponding to the 3’ ends of the viral genome, which were then cloned into the pCR2.1-TOPO vector. Individual plasmid clones corresponded to a single virus variant in the population and were analyzed by agarose gel electrophoresis to distinguish parental and deletion variants emerging during passaging (Fig 2D, top and 2E). Frequencies of emergent viral variants were calculated separately for WT, ΔSLYa, ΔSLYb and ΔSLYab populations (Fig 2D, bottom). As expected, after one passage in mammalian cells (P1), 3’UTR-deletion variants emerged in the WT viral population, representing 10% of the analyzed clones. Interestingly, 3’UTR-deletion variants also arose from viral mutant populations and after P1 represented 5%, 14% and 3% for ΔSLYa, ΔSLYb and ΔSLYab, respectively, not differing significantly from the WT population (Fig 2D). After five viral passages in BHK cells (P5), the WT-adapted population contained only 12% parental virus, and nearly 88% of emergent variants had 3’UTR deletions. In contrast, the P5 populations derived from SLY-deletion mutants comprised 74%, 79% and 67% parental RNAs for ΔSLYa, ΔSLYb and ΔSLYab, respectively, and a minority of variants with 3’UTR deletions (26%, 21% and 33% for ΔSLYa, ΔSLYb and ΔSLYab, respectively). Consistent with our previous results [20], WT-adapted populations comprised a repertoire of viral variants with deletions in the 3’UTR (Figs 2F and S1). Some deletions comprised the DR3 copies, yielding viral variants that carried a single SLY (clones 1, 2, 5 and 10) or the deletion of both SLYs (clones 3 and 7) in the 3’UTR. Instead, other deletions were outside DR3 and yielded viral variants that retained both SLY structures (clones 9 and 11).

Altogether, our experiments demonstrate that the presence of the 3’ SLY is not necessary for efficient viral replication in mammalian cells. However, less diversity is generated within viral 3’UTRs when CHIKV-Caribbean lacks one or both SLYs.

### Viral RNA recombination via template-switching mechanism occurs in the 3’UTR of CHIKV-Caribbean regardless of the presence of a duplicated SLY

In RNA viruses, the composition of viral populations results from the continuous interplay between the generation of genome variability and the selective pressures imposed by the host [24]. Genome variability arises from the introduction of point mutations by an RNA-dependent-RNA-polymerase (RdRp) lacking proofreading activity and RNA recombination events generating chimeric viral genomes during replication. Our experiments showed that during CHIKV-Caribbean adaptation to mammalian cells, 3’UTR deletion variants emerged in viral populations derived from the WT virus and mutant viruses lacking one or both SLYs in their 3’UTRs. However, the proportion of new 3’UTR variants after host adaptation was much lower for SLY-mutant populations.

**Fig 3:**
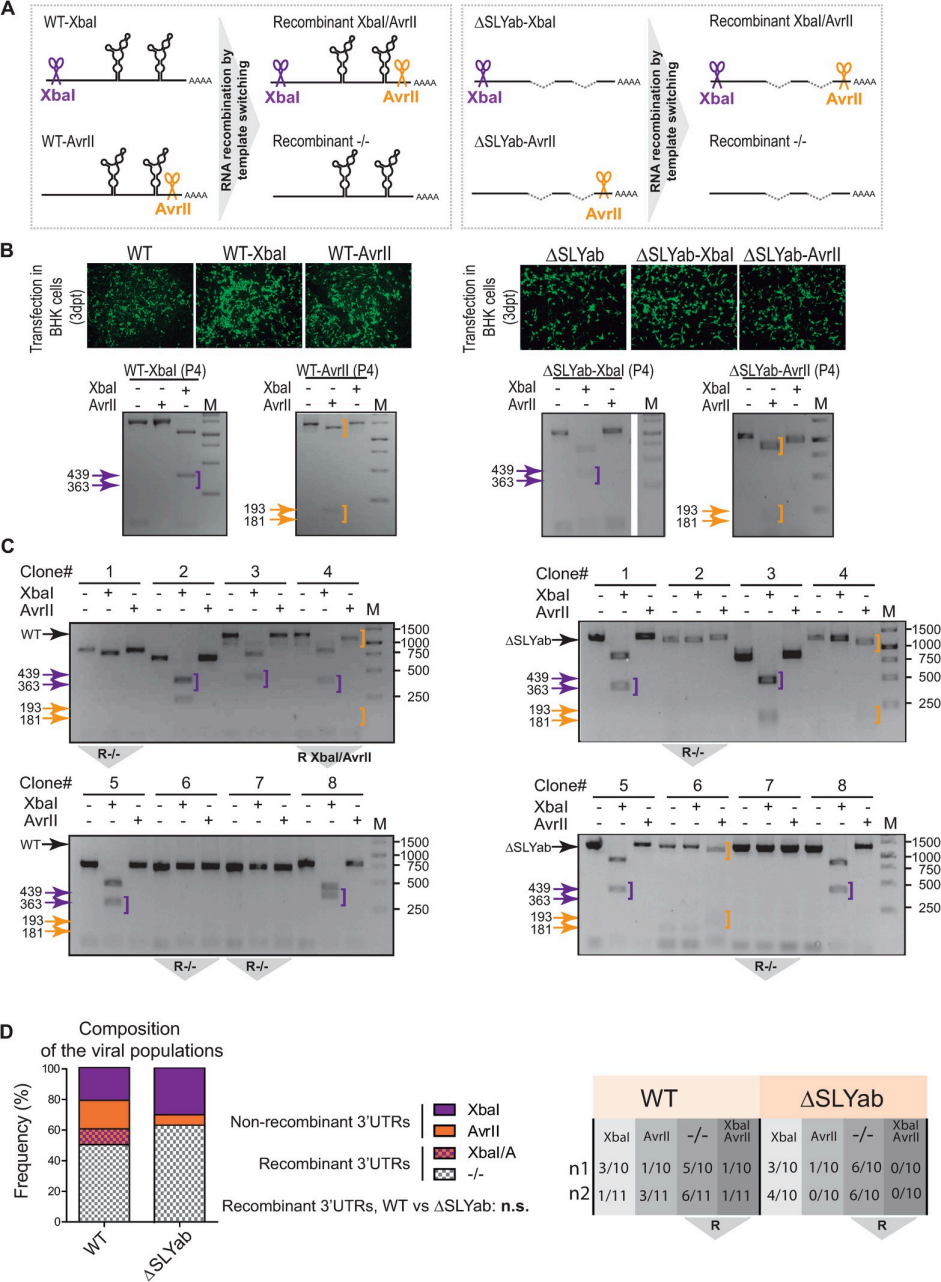
RNA recombination in the CHIKV 3’UTR through template switching between homologous templates. (A) Schematic representation of copy-choice recombination mechanism between WT (top) or ΔSLYab (bottom) viruses differently marked with XbaI (WT-XbaI and ΔSLYab-XbaI) or AvrII (WT-AvrII and ΔSLYab-AvrII) at the 5’ and 3 ‘ends of their 3’UTRs, respectively. After cotransfections, recombinant viruses arise at the 3’UTR (XbaI/AvrII and −/−). (B) Immunofluorescence of WT, WT-XbaI, WT-AvrII, ΔSLYab, ΔSLYab-XbaI, and ΔSLYab-AvrII viruses on day 3 post-transfection of BHK cells (top) and assessment of the presence of XbaI or AvrII marker sites after four successive passages in BHK cells (bottom). Depending on the orientation of each inserted fragment into the blunt plasmid, digestion of XbaI positive clones generated 439 or 363 nt products, and digestion of AvrII positive clones generated 193 or 181 nt products. (C) For illustration, undigested and digested products from eight representative clones are shown for each cotransfection mixture. Recombinant 3’UTR clones are indicated with an inverted gray triangle under each agarose gel. (D) Only clones spanning the full-length fragments were numbered to calculate recombination frequencies. Left, bar graphs showing the relative abundance of recombinant and nonrecombinant full-length 3’UTRs from WT and ΔSLYab viral populations. Nonrecombinant XbaI and AvrII 3’UTR variants are indicated in orange and violet plain colors, respectively, and recombinant XbaI/AvrII and −/− 3’UTR variants are indicated in squared violet/orange and gray/white patterns, respectively. Data were compared with Fisher’s exact test on cumulative data from two biological replicates. Key: ns, not significant. Right, table showing the proportion of 3’UTR variants from two independent experiments (n1 and n2).

We have previously showed that CHIKV 3’UTR-deletion variants are generated by copy-choice recombination of the RNA viral genome through a template-switching mechanism [20]. In this process, RdRp and the nascent strand dissociate from the RNA template, and then reassociate at a different position on another RNA template, guided by sequence homology between blocks of repeated elements. Interestingly, for other RNA viruses, it has been proposed that the presence of RNA structural elements in either the template or the acceptor strand also promotes template switching [25]. Therefore, we reasoned that SLY might function to favor the rate or frequency of RNA recombination by template-switching events in the CHIKV 3’UTR. To test this hypothesis, we developed an approach that allows the detection of chimeric viral RNAs originating through the recombining of two differentially marked viral RNA templates. We introduced point mutations into CHIKV infectious clones to generate recognition sites for two unique restriction enzymes at the 5’ or 3’ end of the 3’UTR. Following this strategy, we obtained two pairs of 5’/3’end marked viral genomes: one for the WT virus and one for the ΔSLYab virus. When 5’ and 3’ marked RNAs for the same virus are mixed and co-transfected into cells, template-switching events can be unequivocally shown by restriction analysis. Viruses with a chimeric 3’UTR will contain either both or none of the marker sites compared to the 3’UTR of viruses present in the original input mixture that contained a single site (Fig 3A). Specifically, we engineered restriction sites for XbaI at position 5–10 after the translation stop codon and for AvrII at position −40–−35 before the poly(A) tail, obtaining the recombinant viruses WT-XbaI, WT-AvrII, ΔSLYab-XbaI and ΔSLYab-AvrII. First, we examined viral replication of the newly marked viruses, finding that all were infective in BHK cells (Fig 3B, top). Importantly, we tested whether the introduced mutations were stable in cell culture at least after four successive passages (Fig 3B, bottom). Culture supernatants were harvested for each recombinant virus from passage four and RNA was extracted. Next, these RNAs were used as templates in reverse transcription-polymerase chain reactions (RT-PCR) amplifying the viral genome’s last 1200 nucleotides. Then, RT-PCR products were cloned into the pCR2.1-TOPO vector. The presence of XbaI or AvrII sites was determined by enzymatic digestion of PCR products amplified from individual clones. XbaI restriction sites in individual clones can be readily assessed by a 439 or 363 nt DNA fragment after digestion of the PCR products with XbaI. Similarly, AvrII restriction sites in individual clones can be assessed by a 193 or 181 nt DNA fragment after digestion of the PCR products with AvrII or more easily, by a shift in the mobility of the remaining fragment. To test the stability of the introduced mutations, we analyzed 10 plasmid clones for WT and ΔSLYab marked viruses, observing that the restriction sites were conserved in all the clones at least after four viral passages. For illustration, digests for one representative viral clone for each WT (AvrII and XbaI) and ΔSLYab (AvrII and XbaI) passaged virus are shown (Fig 3B, bottom).

Next, to evaluate the occurrence of recombination events producing chimeric 3’UTRs in the presence or absence of SLY, equal amounts of RNA transcripts for the WT-XbaI/WT-AvrII and ΔSLYab-XbaI/ΔSLYab-AvrII pairs were separately cotransfected into BHK cells. On day 3 post-transfection, supernatants from both mixtures were collected and used to infect fresh BHK cells. To assess recombination, total RNA was extracted after three days and processed as described above to test the presence or absence of XbaI and AvrII sites in individual plasmid clones (Fig 3C). For WT and ΔSLYab populations, we examined clones containing full-length 3’UTRs and calculated the relative abundances of viral variants. Clones containing either XbaI or AvrII were classified as nonrecombinants, while clones containing XbaI and AvrII sites or no marker sites were classified as recombinants (Fig 3D). Interestingly, about 60% (13 out of 21 total clones with full-length 3’UTR) of viruses recovered from the WT-XbaI/WT-AvrII mixture had recombined in their 3’UTR, and most had no marker sites (11 clones). A similar abundance of RNA recombinants was found with the ΔSLYab-XbaI/ΔSLYab-AvrII mixture (12 out of 20 clones bearing full-length 3’UTR), also with no marker sites (Table in Fig 3D). The fact that variants lacking artificially generated restriction sites vastly outnumbered those with both sites suggested that introducing the restriction sites diminished viral fitness. In fact, competition experiments of the AvrII vs the −/− virus showed that the single nucleotide substitution introduced to generate the AvrII recognition site at the end of the 3’UTR significantly decreased viral fitness. In turn, nucleotide substitutions that generate the XbaI recognition site had no effect on fitness compared to the −/− virus (S2 Fig). While the decreased fitness of the AvrII virus explains the composition of viral populations arising from cotransfection experiments (Fig 3D), it does not impact on the approach to analyze the recombination rate. As noted, the proportions of recombinant 3’UTR variants in the WT and mutant populations were not significantly different. These data indicate that RNA recombination occurs at high rates in the CHIKV 3’UTR, regardless of the presence of the SLY element in DR3, ruling out SLY’s role in favoring recombination. Nevertheless, in vitro evolution experiments (Fig 2D) indicated that, after P5, the WT-adapted population contained more 3’ deletion variants than the SLY-mutant populations. Therefore, we reasoned that the wider spectrum of emerging variants in the WT population was not due to different recombination rates but to strong viral selection processes in mammalian cells. According to fitness parameters (Fig 2B and 2C), it is plausible that new variants lacking DR copies have a replicative advantage over the parental WT virus, explaining their high abundance in the mammalian-adapted population. Conversely, viruses with engineered SLY deletions have per sé high fitness, explaining why in vitro evolution did not result in the selection of new variants with shorter 3’UTRs as observed with the WT virus.

Overall, our findings indicate that the SLY element impacts CHIKV 3’UTR evolution without altering template-switching rates.

### SLY presence in the CHIKV 3’UTR promotes viral replication in mosquito cells

**Fig 4:**
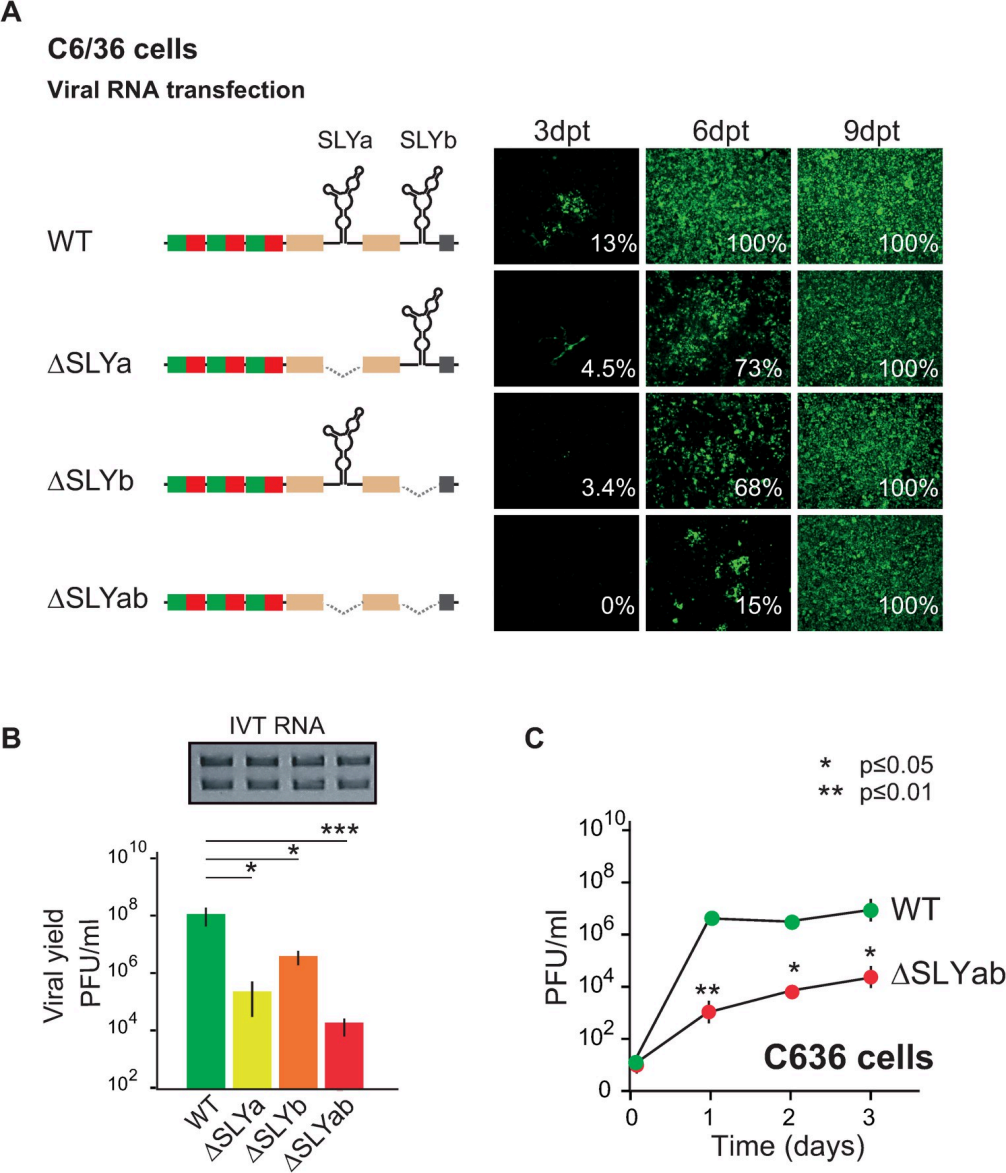
Replication of WT CHIKV and viruses with SLY deletions in mosquito cells. (A) Left, schematic representation of the CHIKV-Caribbean WT virus and mutant viruses with deletions of one or two SLY copies. Right, immunofluorescence staining for WT and mutant viruses in mosquito C6/36 cells on days 3, 6, and 9 post-transfection. Images correspond to one representative experiment out of three biological replicates. The data were analyzed and presented as described in Fig 2. (B) Viral yields in cell culture supernatants on day 6 post-transfection for WT and mutant viruses. The symbols and bars depict the means ± standard deviations of the means from three independent experiments. Data were compared with a two-tailed, unpaired *t*-test. (C) Comparative growth kinetics of WT and ΔSLYab viruses in C6/36 cells. Cells were infected with MOI = 0.1, and data were compared with a two-tailed, unpaired t-test. The symbols and bars depict the means ± standard deviations of the means from two independent experiments.

After finding that SLY is dispensable for efficient viral replication and 3’UTR recombination in mammalian cells but is nevertheless retained in the Asian CHIKV lineages, we next asked whether mosquito-specific selective pressures act on the 3’UTR to maintain SLY elements. To address this question, we evaluated the replication of the WT virus and SLY-deletion mutant viruses (ΔSLYa, ΔSLYb and ΔSLYab) in C6/36 mosquito cells. We observed that deleting one or both SLY copies drastically affected replication in mosquito cells (Fig 4A). On day 3 post-transfection, the WT virus had infected approximately 13% of cells in the monolayer, while the ΔSLYa and ΔSLYb viruses had infected <5%. No ΔSLYab virus infected cells were detected at this time point. On day 6, differences in cell infection percentages were maintained between the WT and SLY-deletion mutant viruses. The WT virus had infected the complete monolayer, while the ΔSLYa, ΔSLYb and ΔSLYab viruses had infected 73%, 68% and 15% of the monolayer, respectively. On day 9, all the mutant viruses had infected the complete monolayer. Differences in viral yields from culture supernatants consistently accompanied differences in growth between the WT virus and SLY-deletion mutant viruses (Fig 4B). For example, on day 6 post-transfection, titers were significantly lower for the ΔSLYa and ΔSLYb viruses (500- and 30-fold) than for the WT virus. However, the virus lacking both SLYs showed the most drastic effect, with viral titers about 7000-fold lower than the WT virus.

To further confirm growth phenotypes, we used stocks of the WT and ΔSLYab viruses to infect fresh C6/36 cells using a multiplicity of infection (MOI) of 0.1 and followed viral replication. The results confirmed that viruses lacking SLY copies showed delayed growth kinetics in mosquito cells compared to the WT virus (Fig 4C). Altogether, these results indicate that the presence of SLY in the CHIKV 3’UTR is necessary for efficient viral replication in mosquito cells, where there is a positive correlation between the number of SLY copies and viral replication. Moreover, our experiments suggest that SLY has a host-dependent role during CHIKV replication: it favors viral replication in mosquito cells but is entirely dispensable in mammalian cells. Furthermore, the duplicated SLY in Asian and Caribbean lineages confers a replicative advantage in mosquito cells.

### Functional characterization of the SLY’s sequence and structural elements

**Fig 5:**
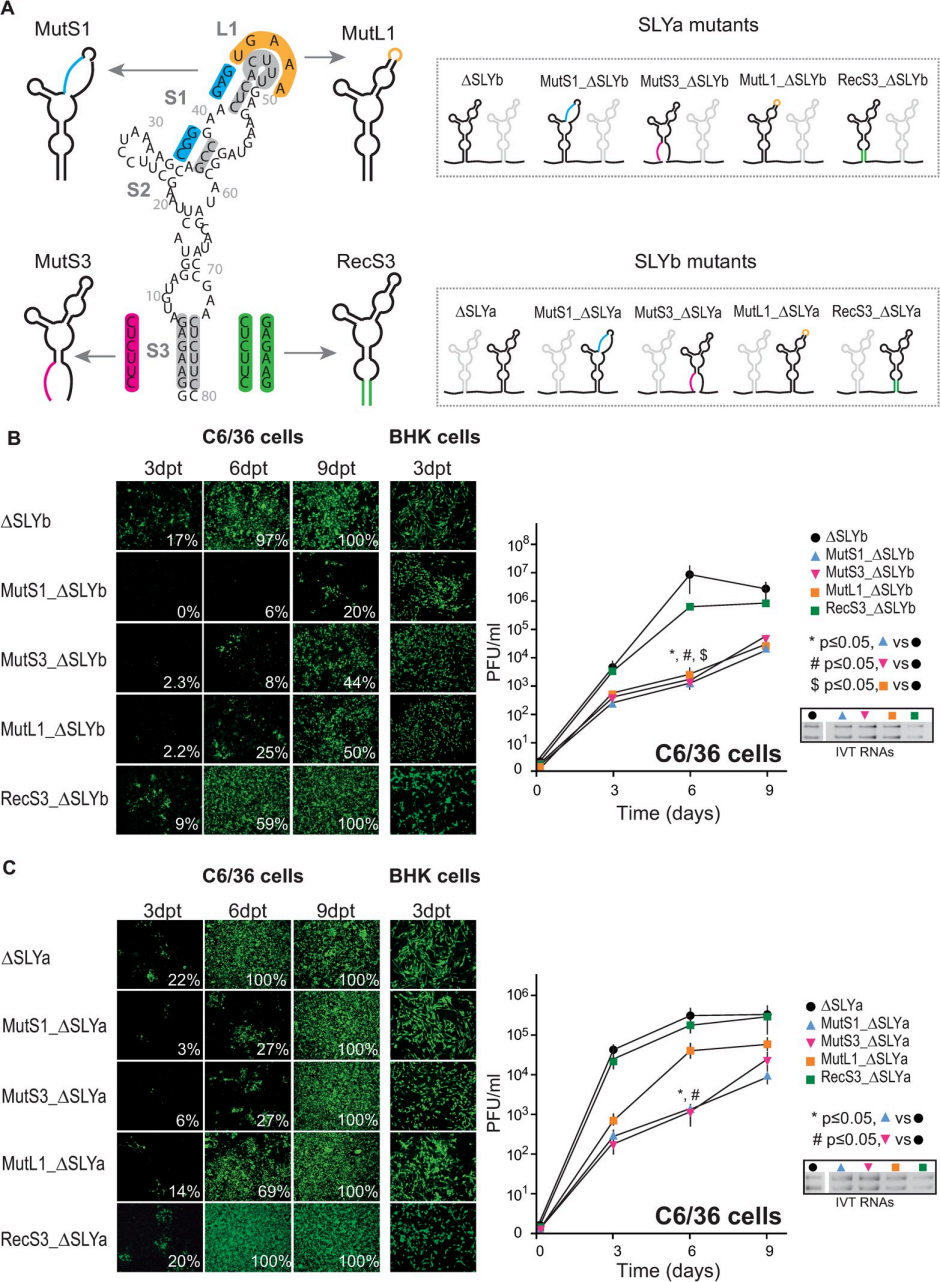
Replication of recombinant viruses with mutations that maintain or disrupt the SLY structure in mosquito cells. (A) Left, schematic representation of the mutations introduced into the SLY in order to generate recombinant viruses that maintain (MutL1 and RecS3) or disrupt (MutS1 and MutS3) its predicted Y-shape RNA structure. In each case, the sequences for WT and mutated SLYs are indicated inside gray and colored boxes, respectively. Right, a schematic representation of the 3’UTRs with SLYa mutations inserted into ΔSLYb (top) and vice versa (bottom). (B and C) Left, immunofluorescences for the mutant ΔSLYb, MutS1_ΔSLYb, MutS3_ΔSLYb, MutL1_ΔSLYb, and RecS3_ ΔSLYb viruses (B) and ΔSLYa, MutS1_ΔSLYa, MutS3_ΔSLYa, MutL1_ΔSLYa, and RecS3_ ΔSLYa viruses (C) in C6/36 and BHK cells at the indicated time points. Images correspond to one representative experiment, with data analyzed as described in Fig 2. Right, viral yields in cell culture supernatants on days 3, 6 and 9 post-transfection. The symbols and bars depict the means ± standard deviations of the means from two independent experiments. Data were compared with a two-tailed, unpaired *t*-test. Agarose gels with IVT RNAs used for transfection in a representative experiment are shown.

According to RNA structure prediction and chemical probing in solution, the SLY contains defined structural elements, including three stems, four bulges, and two loops (Figs 1C and 5A, left). To characterize in detail the role of individual structural elements on viral replication, we designed a panel of recombinant viruses that: (i) modify the L1 sequence without altering the SLY general structure (MutL1); (ii) disrupt the SLY structure by incorporating mismatches into S1’s left strand (MutS1), (iii) disrupt the SLY structure by incorporating mismatches into S3’s left strand (MutS3) and (iv) reconstitute the SLY structure, including artificial nucleotide sequences in both S3 strands (RecS1) (Fig 5A, left). To characterize SLYa and SLYb separately and simplify the experiments’ performance and analyses, we used recombinant viruses containing only one SLY in their 3’UTR (ΔSLYb and ΔSLYa) as the parental genomes. Therefore, we individually introduced each of these mutations into SLYa in the context of the ΔSLYb infectious clone and vice versa (see panels with mutant viruses in Fig 5A, right). We synthesized viral genomic RNA molecules and transfected them in equal amounts (Fig 5B, right) into mosquito and mammalian cells. Next, we evaluated viral replication as a function of time via immunofluorescence assays and viral yields in cell culture supernatants. For SLYa mutants in the ΔSLYb backbone, we found that disrupting S1 drastically impaired viral replication in mosquito cells (Fig 5B, left). In contrast to the parental ΔSLYb virus that was readily detected on day 3 and infected the entire monolayer by day 6, the MutS1_ΔSLYb virus was only detected after day 6 and infected only 20% of the cell monolayer by day 9. Disrupting S3 also significantly delayed viral replication in mosquito cells. MutS3_ΔSLYb replication was barely detectable on day 3 post-transfection and had infected about 8% and 44% of the monolayer on days 6 and 9, respectively. Finally, modifying the L1 sequence also negatively affected viral replication since only 2.2%, 25% and 50% of infected cells were detected on days 3, 6, and 9, respectively. Viral yields consistently followed the immunofluorescence results (Fig 5B, right). On day 6 post-transfection, MutS1_ΔSLYb, MutS3_ΔSLYb and MutL1_ΔSLYb had -8000, -5000 and -3600 fold lower titers than the parental ΔSLYb virus, respectively.

The results suggest that altering the global SLY structure by introducing mismatches greatly impair viral replication in mosquito cells. However, it is possible that the adverse effects result from specific sequence requirements instead of structural constraints. To show that the SLY’s host-specific function relies on the structure and not on the conservation of the nucleotide sequences that pair at the stem’s base, we designed an additional viral mutant carrying an intact SLYa but with a different nucleotide sequence. Briefly, we introduced point mutations into the S3’s right strand in the context of MutS3, which carried changes in the S3’s left strand that disrupted SLYa formation, to reconstitute base pairings and regenerate SLYa folding (RecS3_ΔSLYb, Fig 5A). As expected, the in silico RNA structures folded precisely the same way as the WT virus. Assessment of viral fitness showed that RecS3_ΔSLYb had growth kinetics and reached viral titers comparable to the parental ΔSLYb virus (Fig 5B).

Analogous mutations in SLYb in the context of the ΔSLYa backbone were also tested (Fig 5C). The spread of parental ΔSLYa virus was comparable to ΔSLYb virus. In turn, as observed for the SLYa mutants, mutations disrupting the SLY general structure severely affected viral replication in mosquito cells. MutS1_ ΔSLYa and MutS3_ΔSLYa infected 6% and 27% of cell monolayers on days 3 and 6, respectively, compared to 22% and 100% for the control ΔSLYa virus at the same time points. Mutation of the loop nucleotides (MutL1_ΔSLYa) slightly delayed viral replication, with 14% and 69% of cells infected on days 3 and 6, respectively. Mutations that reconstitute S3 pairings (RecS3_ΔSLYa) infected the entire monolayer on day 6 as the parental ΔSLYa. Viral yields for SLYb mutants recapitulated what we observed when detecting viral antigens. For example, on day 6, MutS1_ ΔSLYa and MutS3_ΔSLYa had about 250-fold lower titers than the parental ΔSLYa virus, MutL1_ΔSLYa only showed a slight decrease in viral titers and RecS3_ΔSLYa reached viral titers comparable to the parental virus (Fig 5C, right).

Finally, we tested whether discrete mutations of SLYa or SLYb in the context of full-length 3’UTR impact viral replication. Mutations disrupting the S3 of SLYa or SLYb were introduced into CHIKV genomes containing both SLYs. Point mutations in either SLY recapitulated our observations with the ΔSLY backbone (S3 Fig). Furthermore, virus yields in the supernatants paralleled the effects of the individual SLY deletions (compare titers on day 4 with those in Fig 4B). Altogether, these results indicate that a duplicated SLY structure is necessary for efficient CHIKV replication in mosquito cells.

Similar analyses were conducted in BHK mammalian cells, where all the mutant viruses could replicate to levels comparable to parental viruses, further confirming that SLY is not required for viral replication in this host (Fig 5B and 5C, middle).

Because mutant virus replication was only evident several days post-transfection, we asked whether the recovered viruses had introduced spontaneous mutations in their 3’UTRs to reconstitute SLY structures. To evaluate this possibility, we performed RT-PCRs and sequencing analyses of viral 3’UTRs on day 9 post-transfection. Interestingly, the engineered sequences of the mutant viruses were retained, and no new changes appeared in the entire 3’UTR region. This result suggests that the recovered viruses can replicate in cell culture with an attenuated viral phenotype. However, we cannot rule out that spontaneous mutations have arisen in other regions of the genome to compensate for defects in viral replication.

In conclusion, the structure of both SLYa and SLYb in the 3’UTR of CHIKV-Caribbean must remain intact for efficient viral replication in mosquito cells. Our results with mutant viruses carrying SLY structures designed with artificial sequences clearly show that the role of this RNA element in mosquito cells depends on its structure and not on the sequence of the base-pairings. The loops’ sequences were also critical for viral replication in mosquito cells, indicating that specific nucleotides should be exposed at the top of SLYs. Interestingly, the effects were more pronounced for SLYa mutants, suggesting that the SLY’s 5’ copy is more sensitive to nucleotide changes.

This is the first report of a host-specialized RNA element in the CHIKV 3’UTR, and supports the view that selective mosquito pressures act on DR3 sequences to maintain SLY’s folding.

### The 3’UTR of distant alphaviruses supports efficient replication of CHIKV in mosquito cells

It has recently been observed that the CHIKV´s replicase can trans-amplify a template RNA carrying the Sindbis virus (SINV) promoter sequences in both mammalian and mosquito cells [26]. In contrast, CHIKV template replication by the SINV replicase is inefficient [26]. A systematic analysis using chimeric templates showed that major determinants for efficient replication by the SINV replicase mapped to the 5’UTR of the RNA template [26].

**Fig 6:**
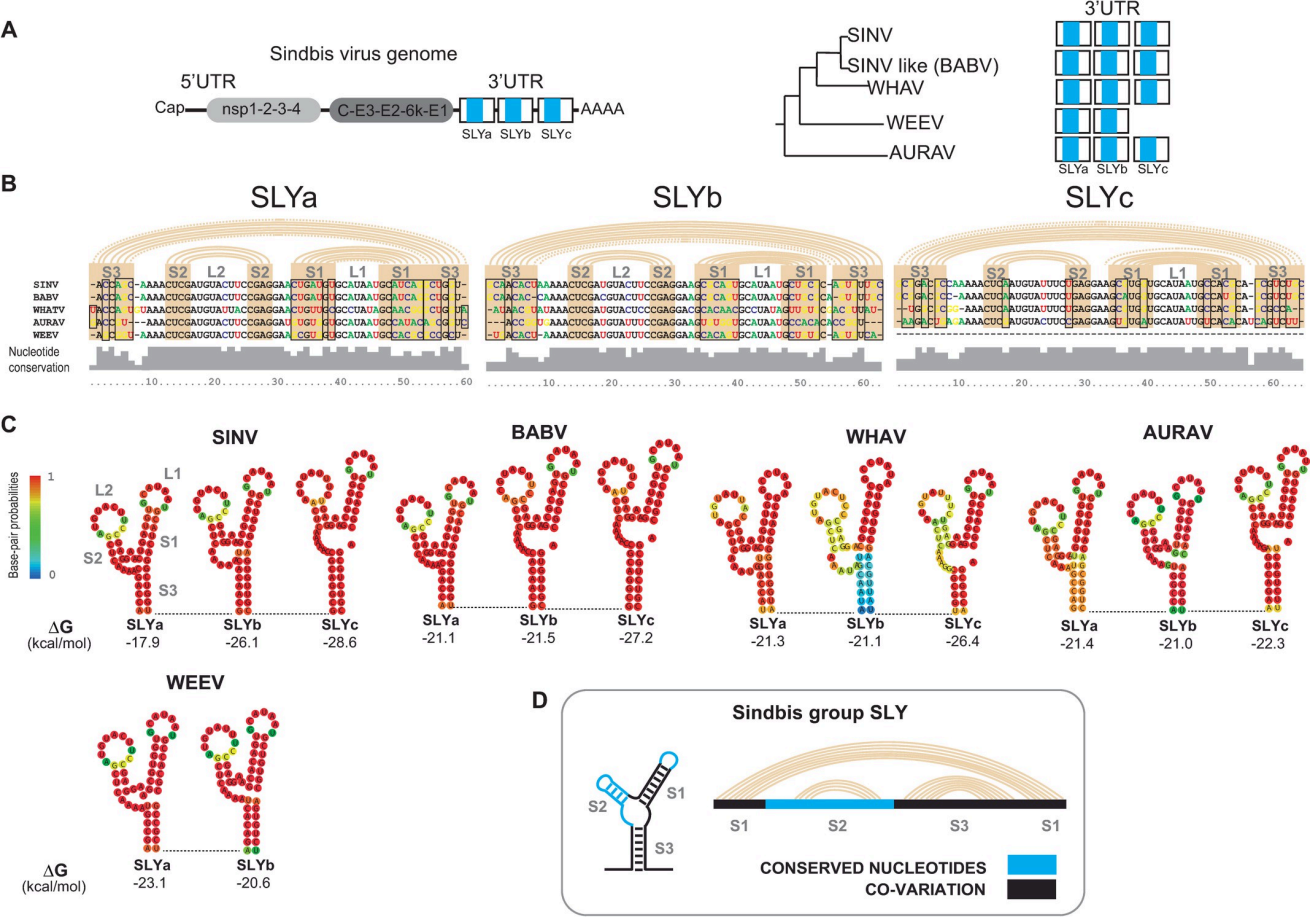
RNA secondary structures in the 3’UTR of Sindbis group viruses. (A) Left, schematic representation of the genomic organization of SINV and its 3’UTR with the RSEs indicated as light blue boxes and regions folding into SLYs in white boxes. Right, phylogenetic relationship between the Sindbis group members (SINV, Babanki ⦍BABV⦎, Whataroa ⦍WHAV⦎, Western equine encephalitis ⦍WEEV⦎, and Aura ⦍AURAV⦎ viruses) and their 3’UTRs. (B) Sequence alignment (middle) and nucleotide conservation plot (bottom, in gray) of the RSEs that fold into SLYa, SLYb, and SLYc for SINV (NC_001547.1), BABV (MF409178.1), WHAV (NC_016961.1), AURAV (NC_003900.1), and WEEV (NC_003908.1). Pink arcs and boxes indicate the nucleotides that pair to form the stems of each Y. Conserved nucleotides are in black, and variable nucleotides are in blue, red, green, and orange. Black boxes indicate the nucleotides that covary among viruses. Key: S, stem; L, loop. (C) Predicted RNA secondary structures for the RSEs in the 3’UTRs of SINV, BABV, WHAV, AURAV and WEEV. Base-pair probabilities are color-coded, and the minimum-free energy for each SLY is shown below. (D) Schematic representation of the SLY regions conserved (in light blue) or covarying (in black) between different SINV group viruses and SLY copies.

SINV is the prototype of the alphavirus genus. Like CHIKV, it alternates between mammalian and mosquito hosts. The 5’ and 3’ ends of its viral genome have been extensively studied [27]. The SINV 3’UTR contains three RSEs copies about 40nt long (Fig 6A, left) that have been predicted to fold into three identical SLYs [28]. Noteworthy, these RNA motifs have a host specific role stimulating translation of SINV genome in mosquito cells [28]. Despite divergence in nucleotide sequences, other members of the SINV group, such as Babanki (BABV), Whataroa (WHAV) and Aura (AURAV) viruses, also contain three copies of the SINV RSE in their 3’UTRs [19]. Moreover, the Western equine encephalitis virus (WEEV), a recombinant virus derived from a SINV-like virus and the Eastern equine encephalitis virus, contains only two RSEs copies (Fig 6A, right) [27]. Considering the common architecture of the 3’UTR of viruses in the SINV group, we wondered whether the SLYs described for SINV were a conserved feature among other group members.

To answer this question, we aligned the 3’UTRs of SINV, BABV, AURAV, WHATV, and WEEV and identified sequences corresponding to RSEs. Next, we individually predicted the RSEs secondary structure, finding that all the members in the SINV group contain 60nt-long regions that fold into highly stable SLYs. The SLY element comprises an upper right stem (S1) that exposes the L1 loop, an upper left stem (S2) that exposes the L2 loop, a central bulge (B1), and a basal stem (S3). Consistent with the number of RSE copies, SLY appears to be triplicated (SLYa, SLYb and SLYc) in SINV, BABV, WHAV, and AURAV and duplicated (SLYa and SLYb) in WEEV. All SLY copies had similar predicted minimum free energies (around -20 kcal/mol) and high base-pair probabilities for individual nucleotides (above 0.8), except those involved in forming S3 in WHAV SLYb (Fig 6C).

The finding of multiple SLY copies in SINV group members prompted us to analyze the nucleotide conservation of SLYa, SLYb, and SLYc together with SLY folding. Interestingly, while nucleotides in L1, S2, and L2 were conserved among species and SLY copies, most nucleotides involved in forming S1 and S3 were highly variable. Strikingly, despite poor nucleotide conservation, base-pair complementarity is fully maintained among copies resulting in structurally identical SLY elements (Fig 6B and 6D). This form of structural conservation, where different nucleotide sequences form similar structures, is known as covariation and provides strong evidence for the formation of functional RNA elements. Here, covariation analysis of the 3’UTRs of SINV and related alphaviruses supports the folding of 2–3 copies of highly stable and conserved SLYs, resembling our analysis with the CHIKV 3’UTR.

**Fig 7:**
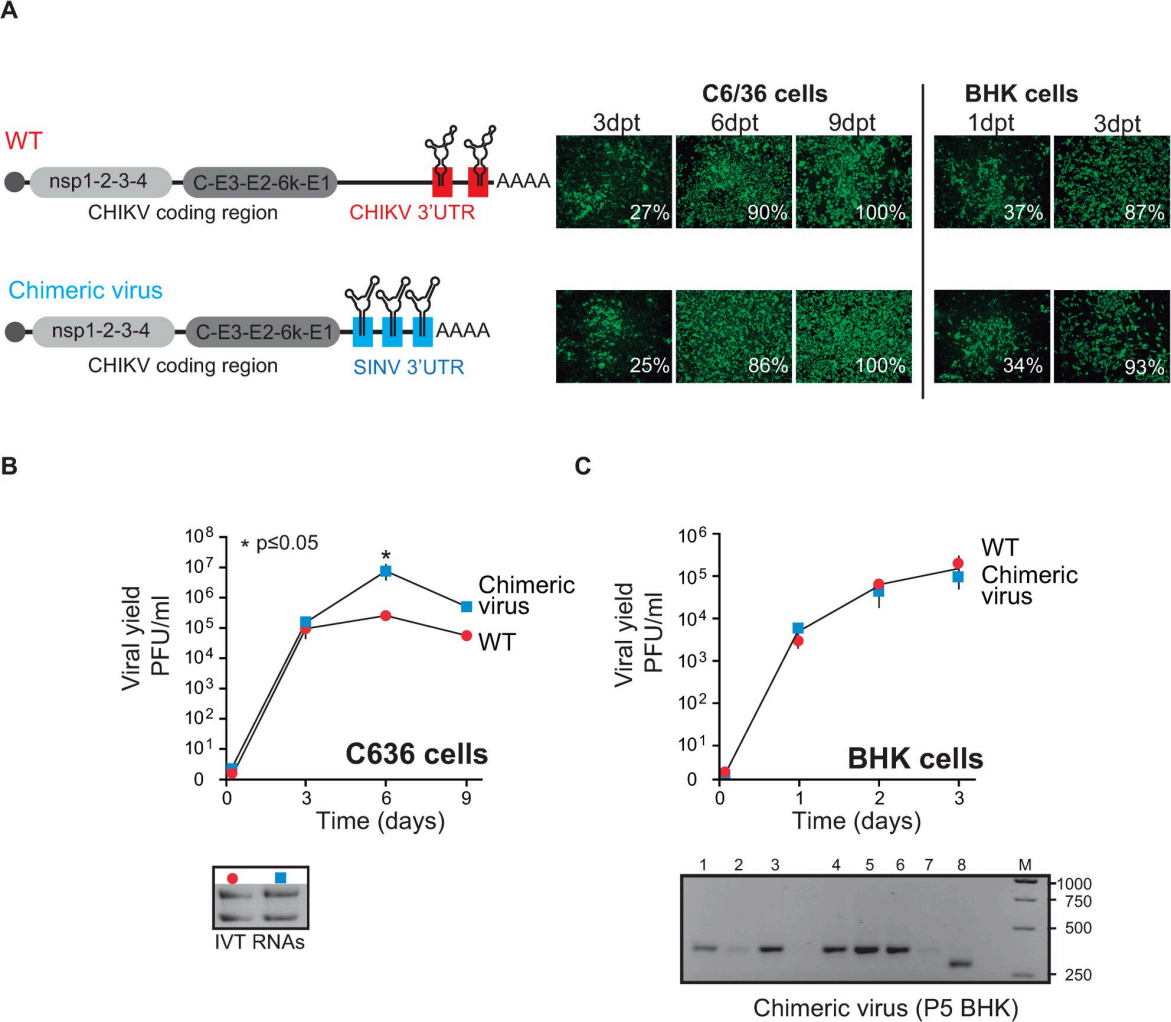
Replication of CHIKV chimeric viruses with the SINV 3’UTR in mosquito and mammalian cells. (A) Left, schematic representation of CHIKV-Caribbean virus and its 3’UTR with two SLY copies (WT) and a chimeric CHIKV- Caribbean virus with the SINV 3’UTR (chimeric virus). Right, immunofluorescence stainings of WT and chimeric viruses in mosquito C6/36 cells on days 3, 6, and 9 post-transfection and in mammalian BHK cells on days 1 and 3 post-transfection. Images correspond to one representative experiment out of two biological replicates. Data were analyzed as described in Fig 2. Viral yields in cell culture supernatants for the WT and the chimeric virus in C6/36 cells (B) and BHK cells (C). The symbols and bars depict the means ± standard deviations of the means from two independent experiments. An agarose gel with IVT RNAs used for transfection. Data were compared with a two-tailed, unpaired *t*-test. Bottom, a representative agarose gel of the PCR amplification of the 3’UTRs of individual chimeric virus clones after P5 in BHK cells. A DNA ladder (M) was used as the reference. The sizes of DNA bands in the ladder (in base pairs) are indicated on the right.

Based on accumulated observations, we asked whether the SINV 3’UTR would function in place of the CHIKV 3’UTR in the context of full-length viruses to support replication in mosquito cells. To address this question, we constructed a viral chimera containing the SINV 3’UTR in the context of the CHIKV-Caribbean genome (Fig 7A, left). Equal amounts of RNA transcripts of parental and chimeric viruses were individually transfected into BHK or C6/36 cells. Next, virus replication was monitored by detecting viral antigens using immunofluorescence assays and quantifying viral yield as a function of time. In both C6/36 and BHK cells, monolayers were infected to a similar extent by parental and chimeric viruses at each time point analyzed (Fig 7A). When we titrated viruses in culture supernatants, we observed that the chimeric virus reached titers about 30-fold higher than the parental virus on day 6 in C6/36 cells (Fig 7B) and similar to the parental virus at the time points tested in BHK cells (Fig 7C). These results show that the SINV 3’UTR was fully functional for viral replication in the CHIKV genomic background.

To assess whether the SINV 3’UTR changed during host adaptation, we performed five successive viral passages in BHK cells and then analyzed the 3’UTRs of the chimeric viral population. Unlike our observations with the CHIKV 3’UTR, which was prone to losing DR copies [20], we found that >85% of the chimeric virus’s progeny contained an intact 323nt-long 3’UTR. These results suggest that the SINV 3’UTR is stable in the CHIKV genome context during adaptation to mammalian cells in cell culture (Fig 7C, bottom panel). Altogether, these data indicate that the SINV 3’UTR can functionally replace the 3’UTR of CHIKV-Caribbean, assuring optimal viral replication in mammalian and mosquito cells.

In summary, in this study we characterized a Y-shaped RNA element in the CHIKV 3’UTR that (i) modulates viral evolution in mammalian cells without altering recombination rate, (ii) assures optimal viral replication in mosquito cells, and (iii) can be replaced by 3’UTRs from distant alphaviruses to replicate efficiently in both hosts. Importantly, SLY’s function in mosquito cells depends highly on its structure and specific top-loop nucleotides.

## Discussion

Here, we describe an RNA structure at the end of the CHIKV’s genome that enhances viral replication in mosquito cells but disfavors viral replication in mammalian cells. Additionally, we found that a duplicated RNA structure in the Asian lineage’s 3’UTR influences the composition of viral population after experimental adaptation, directly impacting the 3’UTR dynamics. Globally, our results constitute the first evidence of a structural RNA element in the CHIKV 3’UTR with a host-specialized function, affecting both viral replication and evolution.

A few RNA secondary structures have already been identified at the ends of alphavirus genomes [6,21,29–31]. In this regard, the alphaviruses’ 5’ UTR has a small RNA element called 5’ stem loop (5’SL) at its very beginning, which is essential for viral RNA synthesis [16,29,32,33] and also interacts with cell factors to evade the host antiviral responses [10]. Nevertheless, due to high heterogeneity in nucleotide sequences and lengths, there is little information on structural RNA elements in the alphaviruses’ 3’ UTR. Insights into the folding of a conserved Y-shaped RNA element (SLY) in the CHIKV 3’UTR were first obtained in silico by us and others [18,20,31]. Here, through a comprehensive analysis of >100 natural CHIKV 3’UTR sequences, we confirmed the presence of the SLY at the end of the CHIKV genome and showed that it varies in copy numbers among lineages. To support our predictions, we contrasted our results with the biochemical probing of the Caribbean virus lineage complete genome in solution, recently published by Madden et al. [21]. While they built a model in which duplicated SLYs do not strictly form at the 3’UTR, experimental nucleotide reactivity fully supports SLYa and SLYb formation. We included reactivity data in our model based on the concept that DRs are blocks of functional elements and fold independently. Our joint analysis showed the folding of a single SLY at the 3’ end of the ECSA/IOL lineages and a duplicated SLY at the 3’ end of the Asian strains.

Duplication of RNA sequences appears to be a common trait among viruses that alternate between hosts with conflicting demands for viral replication. Indeed, it has been suggested that arboviruses have evolved duplicated RNA elements to alleviate the cost imposed by jumping from one host to another. For flaviviruses, there is a clear association between those that alternate between vertebrate hosts and arthropod vectors and the conservation of duplicated RNA structures in their 3’UTR [34]. For alphaviruses, we have recently proposed repeated sequences have been incorporated into their 3’UTR during evolution [35]. In the case of CHIKV, the 3’UTR contains two repeated sequence types [DR(1+2) and DR3] that enhance viral replication in mosquito cells [18,20,23,36]. Importantly, competition experiments in vivo originally established an association between repeated sequences and the efficient virus dissemination [18,20,23,36]. Moreover, we recently provided a precise explanation of the role of an intact 3’UTR during the mosquito life cycle [37]. We showed that a CHIKV-Caribbean mutant missing the three DR(1+2) copies in its 3’UTR had reduced fitness and transmission potential in *Aedes* mosquitoes compared to the WT virus due to an impaired capacity to cross mosquito anatomical barriers [37]. However, an in-depth analysis of the DR3 sequences’ role during virus infection cycle in the mosquito vector is still lacking. Given that RNA secondary structure predictions have shown that DR1 and DR2 do not strictly fold into conserved structural elements but DR3 has a conserved SLY structure, we speculate that DR(1+2) and DR3 are functionally different. Indeed, we have previously shown that while deleting one DR(1+2) copy has a marginal effect on viral replication, deleting one DR3 copy significantly impaired viral replication in mosquito cells [20]. In addition, we showed here that deleting SLY from DR3 had a similar drastic effect on viral replication in mosquito cells to deleting a complete DR3 element, suggesting that the SLY element performs DR3’s role. These observations support the view that DR(1+2) and DR3 may operate through independent mechanisms to enhance viral replication in the insect host.

Using SLY mutants, we showed that efficient viral replication in mosquito cells requires an intact Y-shaped structure in the CHIKV 3’UTR rather than sequence conservation. These findings are consistent with Garcia-Moreno et al., who showed that a triplicated RNA structure in the SINV 3’UTR conferred translatability to genomic and subgenomic mRNAs in mosquito but not in mammalian cells [28]. Moreover, our RNA folding analysis predicts the presence of 2–3 copies of a SLY in the 3’UTR of SINV group members, indicating that RNA structures are a common feature in the 3’UTRs of these alphaviruses. Additionally, our results using mutant viruses with artificially reconstituted SLYs and a chimeric CHIKV with the SINV 3’UTR showed that Y-shaped elements, and not specific sequences involved in base pairings, are important for viral replication in the invertebrate host. Altogether, these observations suggest that defined RNA structures in the alphaviruses’ 3’UTR act as host-specialized elements. Characterization of the SLY’s role during alphavirus infection at a mechanistical level requires further investigation.

Examples of RNA elements that function in a structure-dependent manner are found in many RNA viruses. In some cases, these RNA elements prevent the progress of enzymes or protein/RNA complexes over the viral RNA genome. For instance, the flaviviruses’ 3’UTR contains RNA tertiary structures that resist degradation by the cellular exonuclease Xrn1, leading to the accumulation of one or more species of subgenomic flavivirus RNAs. Xrn1-resistant RNA elements of different flaviviruses vary in primary sequence but form similar tertiary structures, suggesting that they act in a comparable manner and independently of their sequences [38]. Another example is found in the capsid-coding region of dengue and West Nile viruses, where a small RNA hairpin was found to enhance start codon selection during translation [39].

In addition, the stalling of viral replicases induced by structured RNA elements was associated with enhanced RNA recombination rates via template switching in different plant RNA viruses [40] and the human immunodeficiency virus [41]. Since we previously showed that RNA recombination occurs at high rates in the CHIKV 3’UTR, we reasoned that forming of a stable SLY structure at the end of CHIKV genomes may favor template switching. Here, we identified recombination events using WT virus and SLY deletion mutants, finding that a structured SLY did not enhance the template-switching rate. Nevertheless, directed in vitro evolution in mammalian cells indicated that adaptation of the WT virus resulted in a heterogeneous viral population comprising viral variants with diverse 3’UTRs, in contrast to the homogeneous population arising from mutant viruses with one or no SLY at their 3’UTR. Therefore, it is possible that, despite not influencing the recombination rate, the deletion of SLY elements alters the distribution of breakpoints across the 3’UTR, resulting in a distinct mutant spectrum in the virus population after adaptation to mammalian cells. The impact of SLY deletion on population dynamics warrants further investigation.

Diversity is widely accepted as advantageous since it allows the viral population to adapt to new environments and challenges during infection. Furthermore, the cooperative interaction of viral variants can determine pathogenesis in the infected host [42]. Altogether, our results indicate that CHIKV follows different evolutionary pathways in the presence or absence of a duplicated SLY in its 3’UTR. The fact that SLY elements allow CHIKV to exist as a more diverse population might give it the advantage of continuously adapting during the human-mosquito transmission cycle.

Other RNA elements that function in a structure-dependent manner interact with viral or cellular factors to support viral replication. RNA folding is crucial for exposing specific viral genome sequences to the cell environment in these cases. A clear example is found in the 5’UTR of all flavivirus genus members, where an RNA structure named stem-loop A (SLA) acts as the promoter for minus-strand RNA synthesis [3]. The SLA must fold into an intact Y-shaped structure to exert its function since disruption of base pairings completely abolishes the viral polymerase activity. Supporting the SLA structural requirement, base pairing reconstitution with artificial sequences restores the promoter function and fully rescues viral replication. Although an intact SLA structure is necessary and sufficient to bind the viral RNA polymerase, specific nucleotides must be exposed in the top loop to form an active RNA/protein complex and promote de novo RNA synthesis [3]. Consistent with this notion, we found here that it is not only the SLY structure but also the presence of specific nucleotide sequences in the loops that ensures full functionality. In addition, comparative nucleotide analysis of the SINV group’s SLYs showed that the loops are highly conserved among viruses, despite low nucleotide conservation of the overall RNA elements. Strikingly, we found that an ACUU-specific motif in the right loops of CHIKV SLYa and SLYb is also present at least in one of the loops of SINV, BABV, AURAV and WEEV SLYs. Based on this evidence, we favor the hypothesis that the Y-shaped elements in the alphaviruses’ 3’UTR function as nucleotide-presenting structures interacting with a still unknown RNA binding factor within the mosquito host. Altogether, identifying a defined RNA element that differentially modulates viral fitness in a host-specific manner represents new information that explains the host adaptation of an RNA virus.

In summary, this is the first functional study of a conserved RNA secondary structure in the CHIKV 3’UTR with a host-specific function. It sheds new light on understanding different RNA elements comprising the architecture of the alphaviruses’ 3’UTR. Uncovering the functions of viral RNA elements might facilitate our comprehension of virus biology and the requirements for completing efficient transmission cycles.

## Materials and methods

### Sequence alignment and RNA secondary structure prediction

Complete genome sequences of CHIKV natural isolates were obtained from the ViPR database and cured manually to select those with complete 3’UTRs. 112 3’UTR sequences from the East Central and South Africa (ECSA), Indian-Ocean (IOL), and Asian lineages (S1 Table) were aligned using an online version of the Clustal Omega program. The RNA secondary structure predictions were done using the RNAalifold program. The SHAPE constrained thermodynamic model was done with RNAstructure program (version 6.4, Mathews lab) considering the consensus SLYa and SLYb sequences and the SHAPE reactivity values obtained by Madden et al. [21]. Sequences of SINV, Babanki (BABV), Whataroa (WHAV), Aura (AURAV), and Western Equine Encephalitis (WEEV) 3’UTRs were downloaded from GeneBank public database and aligned with Clustal Omega to identify the RSEs. The RSE secondary structures were predicted using the RNAfold program.

### Recombinant CHIKVs

We constructed a 3’UTR cloning cassette spaning from the stop codon of ORF encoding for viral structural proteins to the poly(A) tail to facilitate mutations within the CHIKV-Caribbean 3’UTR infectious clone. The cloning cassette is flanked by SacI and NotI unique restriction sites and allows the exchange of the WT sequence by mutant 3’UTRs. Mutant 3’UTRs of ΔSLYa, ΔSLYb, ΔSLYab, WT-AvrII, ΔSLYab-AvrII, MutS1_ΔSLYb, MutS3_ΔSLYb, MutL1_ΔSLYb, MutS1_ΔSLYa, MutS3_ΔSLYa, MutL1_ΔSLYa, RecS3_ΔSLYb, RecS3_ΔSLYa, MutS3-SLYa, and MutS3-SLYb were generated by overlapping PCRs. The templates and the forward and reverse oligonucleotides used to introduce the specific mutations are given in S2 Table. The overlapping PCRs were performed with the common outside oligonucleotides 5’-TCAGCAGGCACTAAGAGCTCGACAATTAAGTA-3’ and 5’-CGAAACAAGCGCTCATGAGC-3’. The 3’UTRs with XbaI restriction site (WT-XbaI and ΔSLYab-XbaI) were obtained by PCR amplification of the 3’UTR using the sense primer 5’-TCAGCAGGCACTAAGAGCTCTAGAGACAATTAAGTA-3’ and the reverse primer 5’-CGAAACAAGCGCTCATGAGC-3’. The chimeric virus 3’UTR was constructed through PCR amplification using the SINV infectious clone as template, the sense primer 5’-CGAAGATGAGCTCTACGCCCCAATGA-3’, and the reverse primer 5’-TTAGCGGCCGCTTTTTTTTTTTTTTTTTTTTTTTTTTGAAATGT-3’. The PCR products and the CHIKV-Caribbean infectious clone were digested with SacI and NotI restriction enzymes, ligated together and used for transformation of competent E.coli cells. Plasmidic infectious clones were mini-prep purified and Sanger sequenced to verify the presence of the desired mutations.

Recombinant constructs DNAs were linearized by digestion with NotI and used as templates for transcription with SP6 polymerase in the presence of m^7^G(5’)ppp(5’)G cap structure analog, using the mMessage Machine transcription kit (Thermo Fisher). The IVT RNAs were gel-quantified and their integrity was verified by electrophoresis on agarose gels. In each case, equal amounts of RNA transcripts (3 μg/well) were used for transfection in cell culture. Note that IVT reactions result in two RNA products probably due to a cryptic leaky SP6 terminator in the CHIKV-Caribbean infectious clone.

### Cells and viral transfections

Mammalian BHK cells (*Mesocricetus auratus hamster kidney*, ATCC, CCL-10) were grown at 37°C in MEM alpha medium in a 5% CO_2_ atmosphere supplemented with 10% of fetal bovine serum (FBS) and penicillin-streptomycin. Huh-7 cells (*Human hepatocyte cell line*, ATCC, CVCL_0336) were cultured in D-MEM high-glucose medium supplemented with 10% of FBS and penicillin-streptomycin. A primary line of human skin fibroblasts was established at the Instituto de Medicina Translacional e Ingeniería Biomédica [CONICET, Hospital Italiano de Buenos Aires] and grown at 37°C in D-MEM medium.

Mosquito C6/36 cells (*Aedes albopictus*, ATCC, CRL-1660) were grown at 28°C in Leibovitz L-15 medium supplemented with 10% of FBS, 10% of tryptose phosphate, penicillin-streptomycin and amphotericin B. For RNA transfections, cell lines were grown at 60–70% confluence and transfected in 24-well plates using Lipofectamine 2000 (Invitrogen), following manufacturer’s instructions.

### Immunofluorescence assays

BHK and C6/36 cells were seeded in a 24-well plate with a 1cm^2^ coverslip inside and transfected WT or mutant RNAs. Immunofluorescence assays (IF) were performed at different times post-transfection. At each time point, cells were fixed in methanol and stained with a 1:1000 dilution of mouse anti-CHIKV monoclonal antibody NBP2-53111 (Novus) to detect viral antigens. Alexa Fluor 488 goat anti-mouse (Molecular Probes) was used as a secondary antibody at 1:1000 dilution. Estimation of the number of positive cells and calculation of relative percentages of infection were performed with the ImageJ program, using five images for each experimental condition.

### Growth curves

Subconfluent HuH-7, BHK and C6/36 cells and human fibroblasts were seeded in a 24-well plate and infected with equal amounts of WT or mutant viruses. An MOI of 0.1 was used. One hour post-infection, cells were washed five times with PBS and 500 μl of growth media were added. At each time point, cell supernatants were collected and frozen at -70°C. For quantification of infective viral particles, culture supernatants were serially diluted and plaque assays were performed on Vero cells, as described below.

### Plaque assays

Viruses from culture supernatants were quantified by plaque assays to determine viral yields. 10^5^ Vero cells (*Cercopithecus aethiops kidney*, ATCC, CCL-81) were seeded per well in 24-well plates and allowed to attach overnight. After serial dilution, 0.1 ml of cell culture supernatant was added to the wells and incubated for 1 hour at 37°C. Then, 1 ml of overlay (1x D-MEM medium supplemented with 2% of FBS, penicillin-streptomycin, and 0.4% of methylcellulose) was added to each well. On day 3 post-infection, cells were fixed with 10% formaldehyde and stained with crystal violet.

### Experimental host adaptation and sequencing

WT, ΔSLYa, ΔSLYb and ΔSLYab IVT RNAs were transfected into BHK cells in three independent experiments. After two days, viruses were harvested and five successive infections were performed in the same cell line using an MOI of 0.5. Viral RNAs were Trizol-extracted from P1 and P5 culture supernatants and used for RT-PCR reactions with primer 115. Next, PCR reactions were carried out using primers 115 and 116 (5’-CTAATCGTGGTGCTATGC-3). Products were ligated into pCR2.1-TOPO vector and used to transform E.coli XL-1 Blue strain. The total number of clones analyzed for the WT, ΔSLYa, ΔSLYb and ΔSLYab viruses were 52, 20, 21, and 34 for P1; and 69, 31, 33, and 49 for P5, respectively. The lengths of individual viral 3’UTRs were estimated by resolving the 115–116 PCR products in 1.5% agarose gels. For WT P5 populations, 10 individual plasmid clones were sequenced by Sanger method using universal M13R and M13F primers.

### Assessment of recombination after cotransfection of XbaI and AvrII marked RNAs

Two mixtures of in vitro transcribed RNAs were prepared: one with WT-XbaI and WT-AvrII RNAs, and the other with ΔSLYab-XbaI and ΔSLYab-AvrII RNAs. RNA mixtures were separately transfected into BHK cells in two independent experiments. Viral RNAs were Trizol-extracted from culture supernatants on day 3 and used for RT reactions with primer 115. Next, PCR reactions were carried out using primers 115 and 123 (5’-ACTAACGCCGTCACTATTCG-3’). Then, PCR products were cloned into pCR2.1-TOPO vector. A total of 50 and 33 individual clones were analyzed for WT-XbaI/WT-AvrII and ΔSLYab-XbaI/ΔSLYab-AvrII mixtures, respectively. To test XbaI and AvrII restriction sites in individual clones, full-length 3’UTRs were amplified by PCR reactions using universal primers M13F (5’-GTAAAACGACGGCCAGT-3’) and M13R (5’-GTCCTTTGTCGATACTG-3’). Finally, PCR products were digested with XbaI or AvrII restriction enzymes and analyzed by electrophoresis on 1.5% agarose gels. Because the PCR fragments can be inserted with two possible directions, and M13F and M13R primers anneal at different distances from the insertion site in the pCR2.1-TOPO vector, the sizes of the bands generated by digestion depend on directionality of the cloned insert. In addition, the multiple ligation site of the pCR2.1-TOPO vector has an XbaI restriction site, which impacts the size of the XbaI digestion products.

### Growth competition experiments of marked versus unmarked −/− viruses

Viral RNAs of unmarked WT (−/−), WT-XbaI, and WT-AvrII were obtained by IVT and quantified. RNAs were mixed pairwise with ratios of 1 WT (−/−) to 4 WT-XbaI, or 1 WT (−/−) to 4 WT-AvrII. The mixtures were transfected into BHK cells. Viruses were recovered from culture supernantants and used to reinfect fresh cells. After three passages, total RNA was Trizol-extracted from the supernatants and used for RT-PCR reactions. Fragments corresponding to the last ~1200 nucleotides of the viral genome were obtained, cloned into pCR2.1-TOPO vector, digested with XbaI or AvrII restriction enzymes, and analyzed by agarose gel electrophoresis. The relative abundance for each virus in the population was calculated.

## Supporting information

S1 Fig Nucleotide sequences of CHIKV 3’UTRs during adaptation to mammalian cells. Alignment of nucleotide sequences corresponding to the 3’UTR of the WT virus population after five passages (P5) in BHK cells from Fig 2. The input Caribbean WT sequence is presented as the reference. The numbers on the left correspond to those of the clones schematized in Fig 2F. Nucleotide changes are indicated in red. Position 1 refers to the first position after the translation stop codon.
(TIF)

S2 Fig Fitness in mammalian cells of viruses carrying artificially generated restriction sites. (A) Growth curves of WT-XbaI, WT-AvrII and parental WT −/− viruses. BHK cells were infected with MOI = 0.1 and viral titers were estimated by plaque assays (n = 2). (B) Growth competition experiments of marked vs −/− WT virus provides evidence of the decreased fitness of viruses carrying AvrII restriction site in mammalian cells. The relative abundance of each virus is shown in the plot (n = 2).
(TIF)

S3 Fig Viral replication in mosquito cells of recombinant viruses containing the full-length 3’UTR and mutations disrupting the SLY structure. (A) Left, schematic representation of mutants with disrupted S1 of SLYa (MutS3_SLYa) or SLYb (MutS3_SLYb) in the context of a CHIKV genome containing both SLYs. Right, immunofluorescence stainings of WT and mutant viruses in mosquito C6/36 cells on days 4 and 8 post-transfection and in mammalian BHK cells on day 4 post-transfection. Images correspond to one representative experiment out of two biological replicates. Data were analyzed as described in Fig 2. (B) Viral yields in cell culture supernatants for WT and mutant viruses in C6/36 cells. The symbols and bars depict the means ± standard deviations of the means from two independent experiments. An agarose gel with IVT RNAs used for transfection is shown. Data were compared with a two-tailed, unpaired *t*-test.
(TIF)

S1 Table References to CHIKV sequences used in the sequence alignment. (DOCX)

S2 Table PCR oligonucleotides used to construct CHIKV viral mutants. (DOCX)

S3 Table Numerical values that were used to generate the graphs in Figs 2, 4, 5, 7, S2 and S3. (DOCX)

## References

[1] Belshaw R, Pybus OG, Rambaut A. The evolution of genome compression and genomic novelty in RNA viruses. Genome Res. 2007 Oct;17(10):1496–504. doi: 10.1101/gr.6305707 17785537 PMC1987338

[2] Smyth RP, Negroni M, Lever AM, Mak J, Kenyon JC. RNA Structure-A Neglected Puppet Master for the Evolution of Virus and Host Immunity. Front Immunol. 2018 Sep 19;9:2097. doi: 10.3389/fimmu.2018.02097 30283444 PMC6156135

[3] Filomatori CV, Lodeiro MF, Alvarez DE, Samsa MM, Pietrasanta L, Gamarnik AV. A 5’ RNA element promotes dengue virus RNA synthesis on a circular genome. Genes Dev. 2006 Aug 15;20(16):2238–49. doi: 10.1101/gad.1444206 16882970 PMC1553207

[4] Filomatori CV, Iglesias NG, Villordo SM, Alvarez DE, Gamarnik AV. RNA Sequences and Structures Required for the Recruitment and Activity of the Dengue Virus Polymerase [Internet]. Vol. 286, Journal of Biological Chemistry. 2011. p. 6929–39. Available from: doi: http%3A//dx.doi.org/10.1074/jbc.m110.162289 21183683 10.1074/jbc.M110.162289 PMC3044948

[5] Gebhard LG, Filomatori CV, Gamarnik AV. Functional RNA elements in the dengue virus genome. Viruses. 2011 Sep;3(9):1739–56. doi: 10.3390/v3091739 21994804 PMC3187688

[6] Hyde JL, Chen R, Trobaugh DW, Diamond MS, Weaver SC, Klimstra WB, et al. The 5’ and 3’ ends of alphavirus RNAs—Non-coding is not non-functional. Virus Res. 2015 Aug 3;206:99–107. doi: 10.1016/j.virusres.2015.01.016 25630058 PMC4654126

[7] Liu Y, Wimmer E, Paul AV. Cis-acting RNA elements in human and animal plus-strand RNA viruses [Internet]. Vol. 1789, Biochimica et Biophysica Acta (BBA)—Gene Regulatory Mechanisms. 2009. p. 495–517. Available from: doi: 10.1016/j.bbagrm.2009.09.007 19781674 PMC2783963

[8] Wang XW, Liu CX, Chen LL, Zhang QC. RNA structure probing uncovers RNA structure-dependent biological functions [Internet]. Vol. 17, Nature Chemical Biology. 2021. p. 755–66. Available from: doi: 10.1038/s41589-021-00805-7 34172967

[9] Elena SF. RNA virus genetic robustness: possible causes and some consequences [Internet]. Vol. 2, Current Opinion in Virology. 2012. p. 525–30. Available from: 10.1016/j.coviro.2012.06.008 22818515

[10] Reynaud JM, Kim DY, Atasheva S, Rasalouskaya A, White JP, Diamond MS, et al. IFIT1 Differentially Interferes with Translation and Replication of Alphavirus Genomes and Promotes Induction of Type I Interferon. PLoS Pathog. 2015 Apr;11(4):e1004863. doi: 10.1371/journal.ppat.1004863 25927359 PMC4415776

[11] Gamarnik AV, Andino R. Switch from translation to RNA replication in a positive-stranded RNA virus. Genes Dev. 1998 Aug 1;12(15):2293–304. doi: 10.1101/gad.12.15.2293 9694795 PMC317040

[12] de Borba L, de Borba L, Villordo SM, Iglesias NG, Filomatori CV, Gebhard LG, et al. Overlapping Local and Long-Range RNA-RNA Interactions Modulate Dengue Virus Genome Cyclization and Replication [Internet]. Vol. 89, Journal of Virology. 2015. p. 3430–7. Available from: doi: 10.1128/JVI.02677-14 25589642 PMC4337542

[13] Weaver SC. Arrival of chikungunya virus in the new world: prospects for spread and impact on public health. PLoS Negl Trop Dis. 2014 Jun;8(6):e2921. doi: 10.1371/journal.pntd.0002921 24967777 PMC4072586

[14] Puntasecca CJ, King CH, LaBeaud AD. Measuring the global burden of chikungunya and Zika viruses: A systematic review [Internet]. Vol. 15, PLOS Neglected Tropical Diseases. 2021. p. e0009055. Available from: doi: 10.1371/journal.pntd.0009055 33661908 PMC7932082

[15] Kulasegaran-Shylini R, Atasheva S, Gorenstein DG, Frolov I. Structural and functional elements of the promoter encoded by the 5’ untranslated region of the Venezuelan equine encephalitis virus genome. J Virol. 2009 Sep;83(17):8327–39. doi: 10.1128/JVI.00586-09 19515761 PMC2738147

[16] Frolov I, Hardy R, Rice CM. Cis-acting RNA elements at the 5’ end of Sindbis virus genome RNA regulate minus- and plus-strand RNA synthesis. RNA. 2001 Nov;7(11):1638–51. doi: 10.1017/s135583820101010x 11720292 PMC1370205

[17] Kendall C, Khalid H, Mueller M, Kohl A, Merits A, Stonehouse NJ, et al. Structural and phenotypic analysis of Chikungunya Virus RNA structures during viral genome replication and translation [Internet]. Vol. 1, Access Microbiology. 2019. Available from: 10.1099/acmi.ac2019.po0346

[18] Chen R, Wang E, Tsetsarkin KA, Weaver SC. Chikungunya virus 3’ untranslated region: adaptation to mosquitoes and a population bottleneck as major evolutionary forces. PLoS Pathog. 2013 Aug 29;9(8):e1003591. doi: 10.1371/journal.ppat.1003591 24009512 PMC3757053

[19] Pfeffer M, Kinney RM, Kaaden OR. The alphavirus 3’-nontranslated region: size heterogeneity and arrangement of repeated sequence elements. Virology. 1998 Jan 5;240(1):100–8. doi: 10.1006/viro.1997.8907 9448694

[20] Filomatori CV, Bardossy ES, Merwaiss F, Suzuki Y, Henrion A, Saleh MC, et al. RNA recombination at Chikungunya virus 3’UTR as an evolutionary mechanism that provides adaptability. PLoS Pathog. 2019 Apr;15(4):e1007706. 10.1371/journal.ppat.1007706 PMC6502353 30986247

[21] Madden EA, Plante KS, Morrison CR, Kutchko KM, Sanders W, Long KM, et al. Using SHAPE-MaP To Model RNA Secondary Structure and Identify 3’UTR Variation in Chikungunya Virus. J Virol [Internet]. 2020 Nov 23;94(24). Available from: doi: 10.1128/JVI.00701-20 32999019 PMC7925192

[22] Schneider A de B, de Bernardi Schneider A, Ochsenreiter R, Hostager R, Hofacker IL, Janies D, et al. Updated phylogeny of Chikungunya virus suggests lineage-specific RNA architecture [Internet]. Available from: 10.1101/698522 PMC6784101 31470643

[23] Stapleford KA, Moratorio G, Henningsson R, Chen R, Matheus S, Enfissi A, et al. Whole-Genome Sequencing Analysis from the Chikungunya Virus Caribbean Outbreak Reveals Novel Evolutionary Genomic Elements. PLoS Negl Trop Dis. 2016 Jan;10(1):e0004402. doi: 10.1371/journal.pntd.0004402 26807575 PMC4726740

[24] Domingo E, Holland JJ. RNA virus mutations and fitness for survival. Annu Rev Microbiol. 1997;51:151–78. doi: 10.1146/annurev.micro.51.1.151 9343347

[25] Simon-Loriere E, Holmes EC. Why do RNA viruses recombine? Nat Rev Microbiol. 2011 Jul 4;9(8):617–26. doi: 10.1038/nrmicro2614 21725337 PMC3324781

[26] Lello LS, Utt A, Bartholomeeusen K, Wang S, Rausalu K, Kendall C, et al. Cross-utilisation of template RNAs by alphavirus replicases. PLoS Pathog. 2020 Sep;16(9):e1008825. doi: 10.1371/journal.ppat.1008825 32886709 PMC7498090

[27] Strauss JH, Strauss EG. The alphaviruses: gene expression, replication, and evolution [Internet]. Vol. 58, Microbiological Reviews. 1994. p. 491–562. Available from: doi: 10.1128/mr.58.3.491-562.1994 7968923 PMC372977

[28] Garcia-Moreno M, Sanz MA, Carrasco L. A Viral mRNA Motif at the 3’-Untranslated Region that Confers Translatability in a Cell-Specific Manner. Implications for Virus Evolution. Sci Rep. 2016 Jan 12;6:19217. 26755446 10.1038/srep19217 PMC4709744

[29] Nickens DG, Hardy RW. Structural and functional analyses of stem-loop 1 of the Sindbis virus genome. Virology. 2008 Jan 5;370(1):158–72. doi: 10.1016/j.virol.2007.08.006 17900652

[30] Kendall C, Khalid H, Müller M, Banda DH, Kohl A, Merits A, et al. Structural and phenotypic analysis of Chikungunya virus RNA replication elements. Nucleic Acids Res. 2019 Sep 26;47(17):9296–312. doi: 10.1093/nar/gkz640 31350895 PMC6753490

[31] Schneider A de B, Ochsenreiter R, Hostager R, Hofacker IL, Janies D, Wolfinger MT. Updated Phylogeny of Chikungunya Virus Suggests Lineage-Specific RNA Architecture. Viruses [Internet]. 2019 Aug 29;11(9). Available from: doi: 10.3390/v11090798 31470643 PMC6784101

[32] Niesters HG, Strauss JH. Defined mutations in the 5’ nontranslated sequence of Sindbis virus RNA. J Virol. 1990 Sep;64(9):4162–8. doi: 10.1128/JVI.64.9.4162-4168.1990 2384916 PMC247880

[33] Kuhn RJ, Griffin DE, Zhang H, Niesters HG, Strauss JH. Attenuation of Sindbis virus neurovirulence by using defined mutations in nontranslated regions of the genome RNA. J Virol. 1992 Dec;66(12):7121–7. doi: 10.1128/JVI.66.12.7121-7127.1992 1433509 PMC240395

[34] Villordo SM, Carballeda JM, Filomatori CV, Gamarnik AV. RNA Structure Duplications and Flavivirus Host Adaptation. Trends Microbiol. 2016 Apr;24(4):270–83. doi: 10.1016/j.tim.2016.01.002 26850219 PMC4808370

[35] Filomatori CV, Merwaiss F, Bardossy ES, Alvarez DE. Impact of alphavirus 3’UTR plasticity on mosquito transmission. Semin Cell Dev Biol. 2021 Mar;111:148–55. doi: 10.1016/j.semcdb.2020.07.006 32665176

[36] Morley VJ, Noval MG, Chen R, Weaver SC, Vignuzzi M, Stapleford KA, et al. Chikungunya virus evolution following a large 3’UTR deletion results in host-specific molecular changes in protein-coding regions. Virus Evol. 2018 Jan;4(1):vey012. doi: 10.1093/ve/vey012 29942653 PMC6007266

[37] Merwaiss F, Filomatori CV, Susuki Y, Bardossy ES, Alvarez DE, Saleh MC. Chikungunya Virus Replication Rate Determines the Capacity of Crossing Tissue Barriers in Mosquitoes [Internet]. Vol. 95, Journal of Virology. 2021. Available from: doi: 10.1128/JVI.01956-20 33148794 PMC7925089

[38] Kieft JS, Rabe JL, Chapman EG. New hypotheses derived from the structure of a flaviviral Xrn1-resistant RNA: Conservation, folding, and host adaptation. RNA Biol. 2015 Sep 23;12(11):1169–77. doi: 10.1080/15476286.2015.1094599 26399159 PMC4829329

[39] Clyde K, Barrera J, Harris E. The capsid-coding region hairpin element (cHP) is a critical determinant of dengue virus and West Nile virus RNA synthesis. Virology. 2008 Sep 30;379(2):314–23. doi: 10.1016/j.virol.2008.06.034 18676000 PMC2628549

[40] Nagy PD. Recombination in Plant RNA Viruses [Internet]. Plant Virus Evolution. 2008. p. 133–56. Available from: 10.1007/978-3-540-75763-4_8

[41] Simon-Loriere E, Rossolillo P, Negroni M. RNA structures, genomic organization and selection of recombinant HIV. RNA Biol. 2011 Mar;8(2):280–6. doi: 10.4161/rna.8.2.15193 21422815

[42] Vignuzzi M, Stone JK, Arnold JJ, Cameron CE, Andino R. Quasispecies diversity determines pathogenesis through cooperative interactions in a viral population. Nature. 2006 Jan 19;439(7074):344–8. doi: 10.1038/nature04388 16327776 PMC1569948

