# Supplementary figures and images for: A conserved Y-shaped RNA structure in the 3’UTR of chikungunya virus genome as a host-specialized element that modulates viral replication and evolution
Source: PLoS Pathog. 2023 May 1;19(5):e1011352. doi: 10.1371/journal.ppat.1011352 (PMC10174580; doi:10.1371/journal.ppat.1011352)

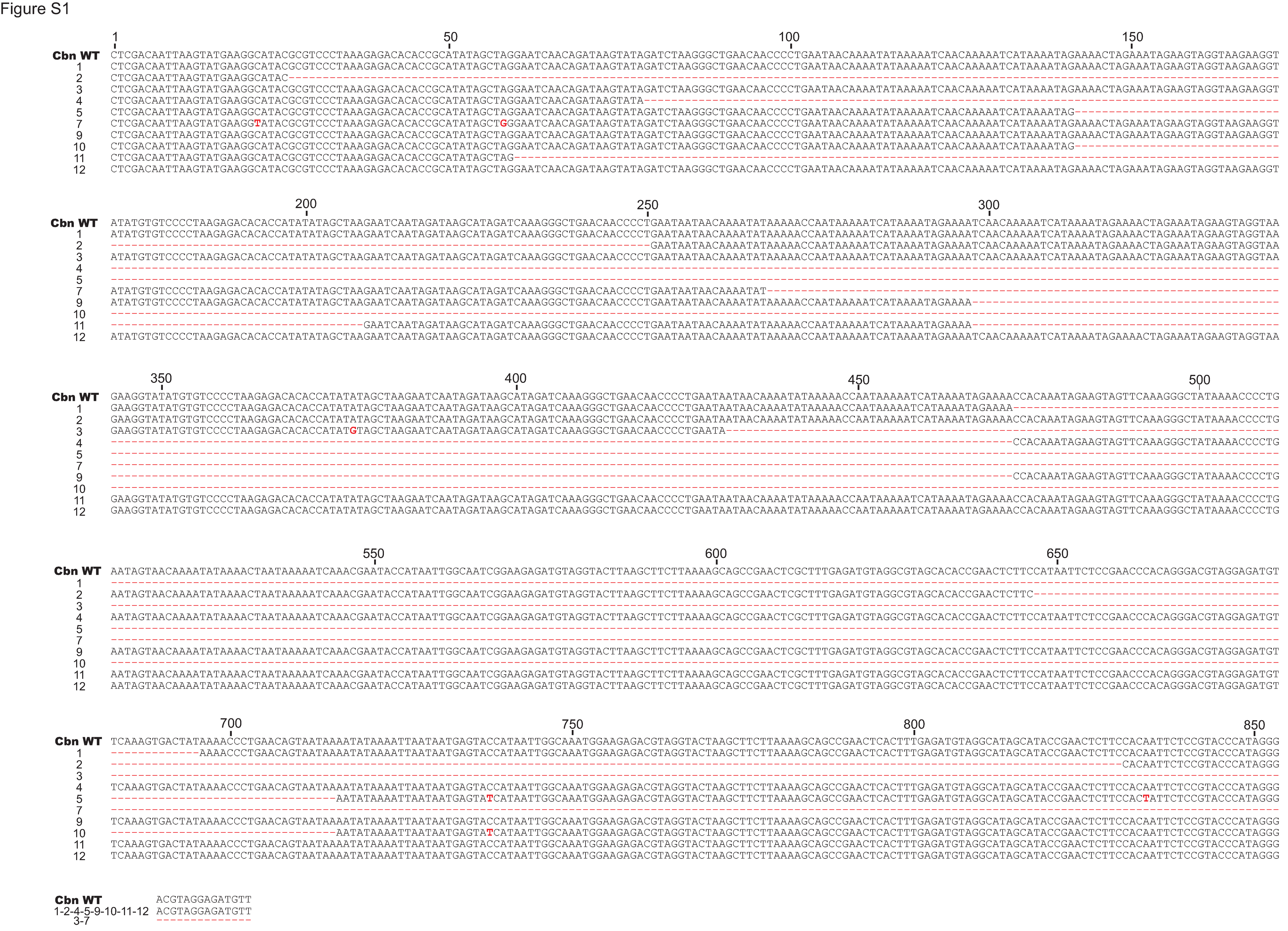

Supplement: S1 Fig — Alignment of nucleotide sequences corresponding to the 3’UTR of the WT virus population after five passages (P5) in BHK cells from Fig 2. The input Caribbean WT sequence is presented as the reference. The numbers on the left correspond to those of the clones schematized in Fig 2F. Nucleotide changes are indicated in red. Position 1 refers to the first position after the translation stop codon. (TIF) [file ppat.1011352.s001.tif]

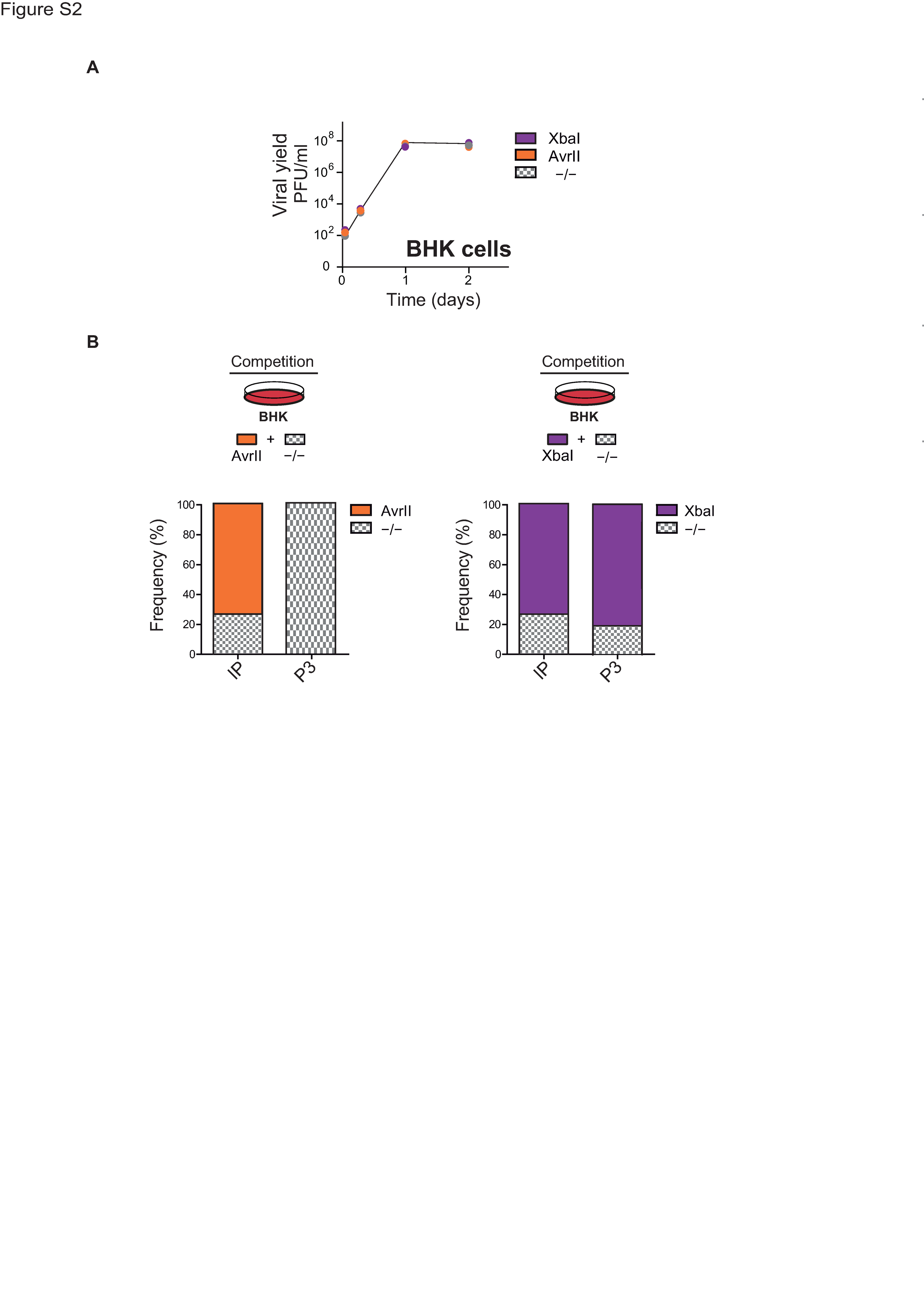

Supplement: S2 Fig — (A) Growth curves of WT-XbaI, WT-AvrII and parental WT −/− viruses. BHK cells were infected with MOI = 0.1 and viral titers were estimated by plaque assays (n = 2). (B) Growth competition experiments of marked vs −/− WT virus provides evidence of the decreased fitness of viruses carrying AvrII restriction site in mammalian cells. The relative abundance of each virus is shown in the plot (n = 2). (TIF) [file ppat.1011352.s002.tif]

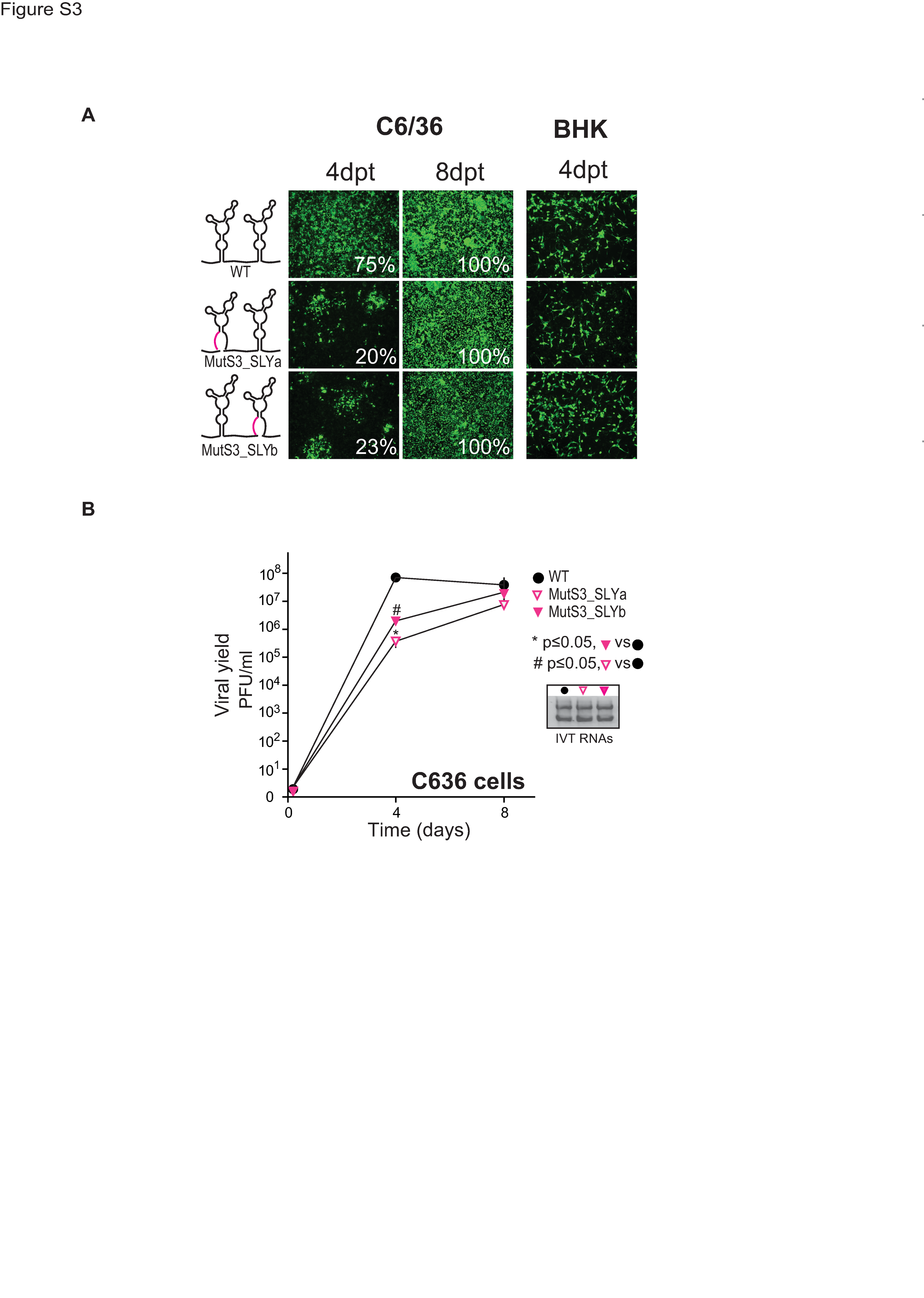

Supplement: S3 Fig — (A) Left, schematic representation of mutants with disrupted S1 of SLYa (MutS3_SLYa) or SLYb (MutS3_SLYb) in the context of a CHIKV genome containing both SLYs. Right, immunofluorescence stainings of WT and mutant viruses in mosquito C6/36 cells on days 4 and 8 post-transfection and in mammalian BHK cells on day 4 post-transfection. Images correspond to one representative experiment out of two biological replicates. Data were analyzed as described in Fig 2. (B) Viral yields in cell culture supernatants for WT and mutant viruses in C6/36 cells. The symbols and bars depict the means ± standard deviations of the means from two independent experiments. An agarose gel with IVT RNAs used for transfection is shown. Data were compared with a two-tailed, unpaired t-test. (TIF) [file ppat.1011352.s003.tif]
