# Supplementary material for: A conserved Y-shaped RNA structure in the 3’UTR of chikungunya virus genome as a host-specialized element that modulates viral replication and evolution
Source: PLoS Pathog. 2023 May 1;19(5):e1011352. doi: 10.1371/journal.ppat.1011352 (PMC10174580; doi:10.1371/journal.ppat.1011352)
Supplement: S2 Table — (DOCX) [file ppat.1011352.s005.docx]

**S2 Table. PCR oligonucleotides used to construct CHIKV viral mutants.**

| **Mutant virus** | **Template** | **Oligonucleotide used for PCRs** |
| --- | --- | --- |
| ΔSLYa | WT | F CCATAATTGGCAAACATTCTCCGAACCCA  R TGGGTTCGGAGAATGTTTGCCAATTATGG |
| ΔSLYb | WT | F CCATAATTGGCAAACATTCTCCGAACCCA  R TGGGTTCGGAGAATGTTTGCCAATTATGG |
| ΔSLYab | ΔSLYa | F CCATAATTGGCAAACATTCTCCGAACCCA  R TGGGTTCGGAGAATGTTTGCCAATTATGG |
| WT-XbaI | WT | F TCAGCAGGCACTAAGAGCTCTAGAGACAATTAAGTA |
| WT-AvrII | WT | F ATTCTCCGTACCCCTAGGGACGTAGGAGATG  R CATCTCCTACGTCCCTAGGGGTACGGAGAAT |
| ΔSLYab-XbaI | ΔSLYab | F TCAGCAGGCACTAAGAGCTCTAGAGACAATTAAGTA |
| ΔSLYab-AvrII | ΔSLYab | F ATTCTCCGTACCCCTAGGGACGTAGGAGATG  R CATCTCCTACGTCCCTAGGGGTACGGAGAAT |
| MutS1_ΔSLYb | ΔSLYb | F AAAGCACGGGAGGCTTTGAGATGTAGGCG  R CAAAGCCTCCCGTGCTTTTAAGAAGCTT |
| MutS3_ΔSLYb | ΔSLYb | F CAATCGCTTCTCATGTAGGTACTTAAGCTTC  R CTACATGAGAAGCGATTGCCAATTATGGTA |
| MutL1_ΔSLYb | ΔSLYb | F GCCGAACTCTGAAAGAGATGTAGGCGTAG  R CTACATCTCTTTCAGAGTTCGGCTGCTTTTAAGAAG |
| MutS1_ΔSLYa | ΔSLYa | F AAAGCACGGGAGGCTTTGAGATGTAGGCG  R CAAAGCCTCCCGTGCTTTTAAGAAGCTT |
| MutS3_ΔSLYa | ΔSLYa | F CAATCGCTTCTCATGTAGGTACTTAAGCTTC  R CTACATGAGAAGCGATTGCCAATTATGGTA |
| MutL1_ΔSLYa | ΔSLYa | F GCCGAACTCTGAAAGAGATGTAGGCGTAG  R CTACATCTCTTTCAGAGTTCGGCTGCTTTTAAGAAG |
| RecS3_ΔSLYb | MutS3_ΔSLYb | F ACCGAAGAGAAGCATAATTCTCCGAACCCA  R ATTATGCTTCTCTTCGGTGTGCTACGCCTAC |
| RecS3_ΔSLYa | MutS3_ΔSLYa | F ACCGAAGAGAAGCATAATTCTCCGAACCCA  R ATTATGCTTCTCTTCGGTGTGCTACGCCTAC |
| MutS3_SLYa | WT | F CAATCGCTTCTCATGTAGGTACTTAAGCTTC  R CTACATGAGAAGCGATTGCCAATTATGGTA |
| MutS3_SLYb | WT | F CAATCGCTTCTCATGTAGGTACTTAAGCTTC  R CTACATGAGAAGCGATTGCCAATTATGGTA |
