# Supplementary material for: A conserved Y-shaped RNA structure in the 3’UTR of chikungunya virus genome as a host-specialized element that modulates viral replication and evolution
Source: PLoS Pathog. 2023 May 1;19(5):e1011352. doi: 10.1371/journal.ppat.1011352 (PMC10174580; doi:10.1371/journal.ppat.1011352)
Supplement: S3 Table — (DOCX) [file ppat.1011352.s006.docx]

**S3 Table. Numerical values that were used to generate the graphs in Figs 2, 4, 5, 7, S2 and S3.­­­**
